# Mg^2+^ Ions as Interfacial Charge Bridges for Enhanced Photocatalytic Hydrogen Evolution in Poly(Heptazine Imide)

**DOI:** 10.1002/smll.75403

**Published:** 2026-08-27

**Authors:** Gabriel A. A. Diab, Vitor G. S. Pastana, Sara Stolfi, Rayse M. Ferreira, Izadora F. Reis, Marcos A. R. da Silva, Caio V. C. Ribeiro, Kathrin Küster, Piero Torelli, Paolo Ghigna, Viola Duppel, Maurizio Fagnoni, Sebastian Bette, Bruna R. Serino, Andre F. de Moura, Davide Ravelli, Bettina V. Lotsch, Ivo F. Teixeira

**Affiliations:** ^1^ Chemistry Department Federal University of São Carlos São Carlos São Paulo Brazil; ^2^ Nanochemistry Department Max Planck Institute for Solid State Research Stuttgart Germany; ^3^ Department of Chemistry University of Pavia Viale Taramelli Pavia Italy; ^4^ Istituto Officina dei Materiali TASC Laboratory CNR‐IOM, Trieste Friuli‐Venezia Giulia Italy; ^5^ Interface Analysis Max Planck Institute for Solid State Research, Stuttgart Baden Württemberg Germany

**Keywords:** carbon nitrides, crystalline carbon nitrides, hydrogen evolution reaction (HER), in situ x‐ray absorption spectroscopy (XAS), poly(heptazine imide) (PHI), synergistic production boost

## Abstract

Optimizing charge transfer at the solid‐liquid interface remains a central challenge for photocatalytic hydrogen evolution, particularly in polymeric semiconductors with limited electron‐donating ability. Here, we demonstrate that Mg^2+^ incorporated into poly(heptazine imide) (PHI) acts as a catalytic promoter by synergistically coupling semiconductor charge transport with Pt co‐catalysts, thereby overcoming kinetically limited interfacial electron transfer. Using H_2_ evolution as a model reaction, Mg incorporation into Na‐PHI enhanced the hydrogen evolution rate by more than 2.5‐fold and introduced a linear dependence on Pt loading, an effect absent in pristine Na‐PHI. In situ XANES reveals reversible electronic modulation of Mg^2+^ exclusively in the presence of Pt and water, confirming its direct participation in interfacial charge mediation. Complementary photophysical, (photo)electrochemical, and molecular dynamics simulations indicate that Mg^2+^ polarizes water molecules through a dynamic Mg^2+^‐OH^δ–^H^δ+^‐Pt interfacial motif, facilitating proton transfer toward Pt while stabilizing hydroxide species. Beyond the activity enhancement, Mg‐PHI exhibits improved structural stability by resisting framework protonation during recycling. Collectively, these findings establish Mg^2+^ as a catalytic promoter and demonstrate that interfacial charge‐bridge effects can be rationally heterogenized, providing a general strategy for enhancing photocatalytic hydrogen evolution through targeted interface engineering.

## Graphical abstract

Mg^2+^ confined within poly(heptazine imide) acts as a heterogenized interfacial charge bridge capable to couple synergistically the semiconductor with Pt co‐catalysts, thus enhancing hydrogen evolution. Through water polarization and hydroxide stabilization, a dynamic Mg^2+^‐OH^δ−^‐H^δ+^‐Pt motif facilitates proton transfer and overcomes interfacial electron‐transfer bottlenecks in photocatalytic hydrogen evolution.

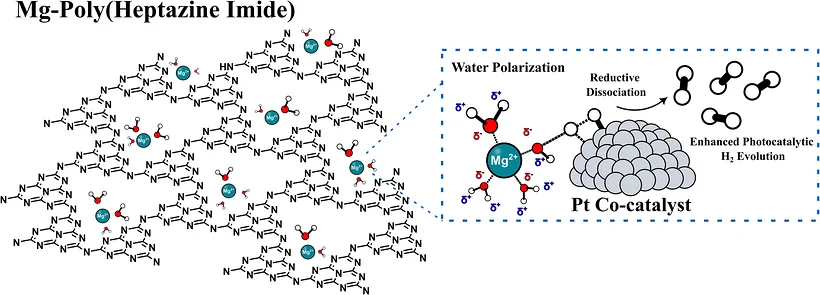

## 1 Introduction

Facing the current energy crisis, H_2_ has emerged as a crucial pillar for the upcoming energy transition, playing a pivotal role in the pursuit of carbon neutrality. As a versatile energy vector, hydrogen incorporates several fundamental principles of green chemistry when employed as an energy carrier, while simultaneously offering multifunctionality across diverse applications. In addition to its long‐standing use as a feedstock for essential industrial processes, hydrogen is increasingly applied in fuel cells, energy storage, transportation, and heating [1, 2]. Despite these advantages, hydrogen still faces significant challenges, particularly concerning its storage, distribution, and, most critically, its production. Currently, approximately 98% of global H_2_ production depends on fossil fuels‐based processes, mainly steam methane reforming and coal gasification, which together account for nearly 900 million metric tons of CO_2_ emitted annually [1].

The search for cleaner and renewable hydrogen production methods has motivated substantial global research efforts. Among the various approaches, solar‐driven photocatalysis represents one of the most attractive pathways. In this field, following the demonstration of the ability of carbon nitrides to split water driven by light, this class of materials has rapidly become one of the most studied semiconductors for different photocatalytic applications [3, 4, 5, 6]. Carbon nitrides are organic polymers built of condensed aromatic rings based on nitrogen and carbon, arranged alternatively to form a heptazine core as their fundamental building block. In the polymeric structure, this monomer is connected by an imide bridge extending it into two‐dimensional sheets stacked in a ladder‐like structure, akin to graphene. This class of materials is recognized for being non‐toxic, chemically and physically stable and easy to fabricate at low cost [7]. Additionally, the associated semiconductor properties, accessible upon visible‐light irradiation, open the door to a broad spectrum of applications, particularly in chemical and energy photoconversion [8]. Among the different crystalline structures within the carbon nitride family, poly(Heptazine‐Imide) (PHI) materials have attracted particular attention. These materials are characterized by a two‐dimensional arrangement of heptazine units interconnected through negatively charged imide nitrogen bridges, thus resulting in an ionic framework [6]. This spatial organization gives rise to periodically distributed triangular pores filled with, typically, alkali ions as compensation cations. The higher degree of periodicity within PHI frameworks facilitates the mobility of photogenerated charges, thereby leading to superior photocatalytic performance compared with the more extensively studied polymeric carbon nitride (PCN—also known as melon or *g*‐C_3_N_4_) [6, 9]. The ionic character renders PHIs highly adaptable to electronic modifications by altering the compensating metal, thereby broadening their functional versatility [7, 10].

In this context, carbon nitrides in general, including those with ordered structure, still face intrinsic limitations hindering their performance. Among these challenges, the high rate of exciton recombination and the kinetic constraints associated with transferring photogenerated charges to the reaction medium are particularly significant, as these materials are thermodynamically poor electron donors [11]. To address these limitations, co‐catalysts, typically noble metals such as Pt, Au, Ir, and Rh, are often employed. These co‐catalysts are well known for lowering the overpotential required for water reduction and for suppressing electron‐hole recombination by efficiently capturing photogenerated electrons, thereby enhancing the overall photocatalytic efficiency [12]. Nevertheless, although these metals are highly efficient for the adsorption process and subsequently aggregating hydrogen species on their surface, they are ineffective at promoting cleavage of the H─OH bond in water molecules. This limitation represents a critical bottleneck for such systems [13].

In parallel, recent advances in interfacial chemistry have revealed that hydrated alkali and alkaline‐earth cations can significantly enhance diverse chemical reactions in both electrochemical and photochemical systems. Owing to their high charge density, these cations polarize water molecules within their hydration shell [14, 15], a phenomenon that can be exploited to accelerate reaction steps involving water‐derived intermediates‐including the elementary steps of the hydrogen and oxygen evolution reactions [15]. Notably, the magnitude of this polarization effect correlates with the hydration energy of the cation [16, 17]. Building on this concept, subsequent studies have reported enhanced catalytic performance in the presence of alkali/alkaline‐earth cations dissolved in the reaction medium [15, 16, 18, 19]. Despite these recurring observations, the pioneering studies establishing this effect were limited to systems in which the cations were homogeneously dispersed in solution. To date, no systematic investigations have been made to “heterogenize” this phenomenon, i.e., to stabilize such cations within a solid matrix while preserving their ability to induce water polarization and promote catalytic activity. Importantly, this strategy would not only reduce the amount of metal precursors required, since the cations would be incorporated solely during material synthesis, allowing their reuse instead of their loss in solution, but would also enable direct investigation of how these ions operate at the solid‐liquid interface, thereby offering unprecedented insight into their mechanistic role in catalytic processes.

We address this gap by investigating the incorporation of Mg^2+^ into the PHI framework and its impact on photocatalytic hydrogen evolution. Mg^2+^ was selected due to its high hydration energy, which is expected to induce a strong water polarization effect, thereby facilitating a more direct and unambiguous investigation of its mechanistic contribution to the photocatalytic process. Starting from Na‐PHI as a parent structure, we achieved controlled Mg^2+^ insertion, which allowed for a systematic evaluation of its role in the photocatalytic reaction. Indeed, our results demonstrate that Mg^2+^ within the framework operates as a catalytic promoter in the HER. By incorporating only 5.5% of Mg^2+^, the final material was able to significantly boost hydrogen evolution, with rates approaching a threef‐old increase, particularly when coupled with Pt co‐catalysts. Through a combination of photocatalytic and (photo)electrochemical essays, computational simulations and in situ XANES, we uncover that Mg^2+^ serves as an interfacial catalytic promoter, acting as a charge mediator between the PHI support and the Pt nanoparticles, demonstrating a novel interfacial electronic interaction but also resolve the long‐standing ambiguity regarding the role of alkaline‐earth metal doping in these systems.

## 2 Results and Discussion

### 2.1 Material's Characterization

The bottom‐up fabrication of the PHI material was achieved through the thermal polycondensation of melamine as a nitrogen rich precursor to obtain the polymeric backbone [16, 20]. Concerning the different PHI arrangements reported in the literature, sodium‐containing poly(heptazine imide) (Na‐PHI) was selected due to its well‐defined crystalline structure and versatility to stabilize different cations, making it the most suitable framework for the study [6, 21, 22]. In contrast to other approaches previously described, wherein the attainment of well‐ordered 2D long‐range heptazine units typically depends on a molten salt medium (such as eutectic mixtures of alkali salts, KSCN and others), the Na‐PHI fabrication utilizes solely NaCl, which remains in the solid state during PHI formation (Scheme 1) [23, 24, 25]. In the final ordered structure, sodium cations introduced are hosted within the triangular pores of the PHI near the bridging imide nitrogen atoms, serving as a counter‐ion throughout the plane, conferring a symmetrical distribution of the pores along the in‐plane domain. The Na^+^ cations present in the structure can be readily replaced by Mg^2+^ through a straightforward cation exchange procedure in an aqueous solution [10, 26]. While this strategy has been extensively employed to modulate the electronic and catalytic properties of PHI using alkali and transition metals, often yielding single‐atom sites, the incorporation of alkaline‐earth metals remains poorly explored [7, 27, 28, 29, 30].

**SCHEME 1:**
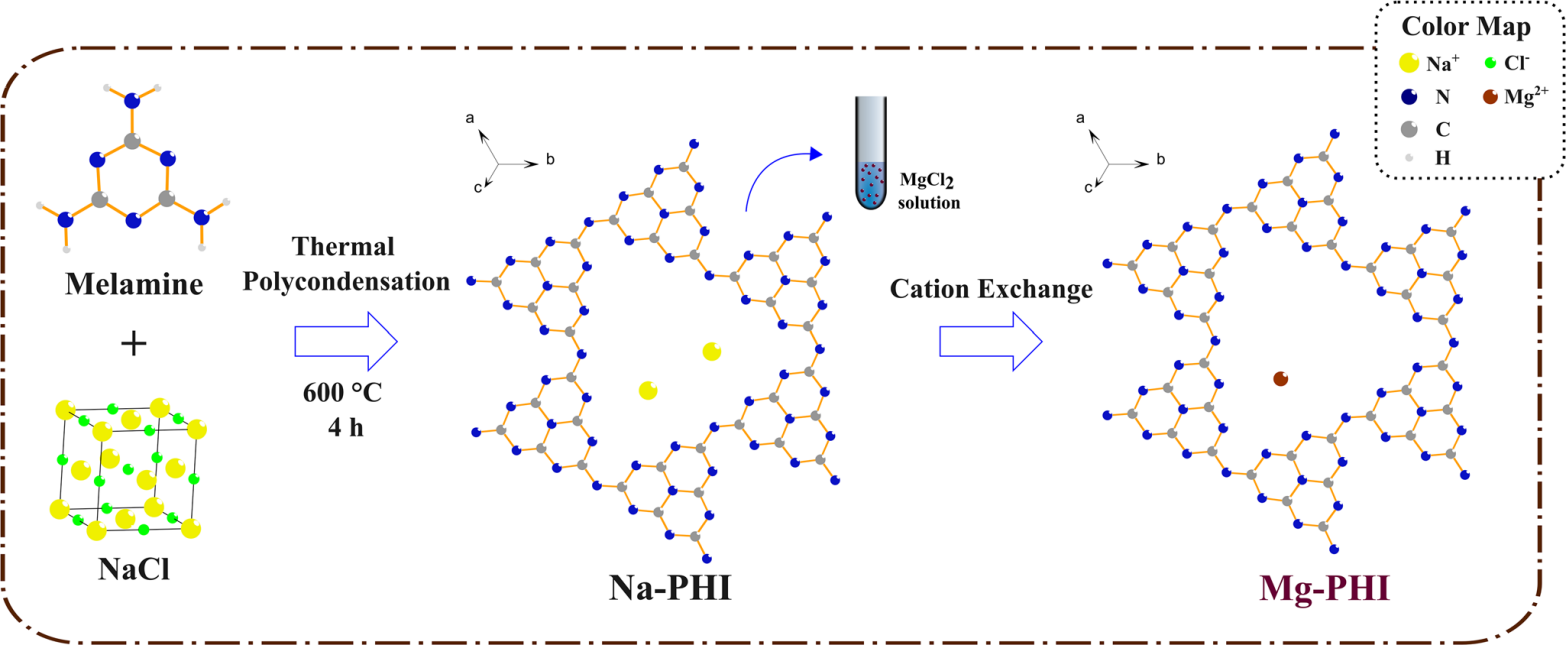
Illustrative representation of the Mg‐PHI fabrication. Initially, the original Na‐PHI is formed by a bottom‐up synthesis using melamine and NaCl as starting materials. Once the Na‐PHI is formed, the Na^+^ ions are replaced by Mg^2+^ through a cation exchange in aqueous medium. Color map: dark gray—C, green—Cl, blue—N, light gray—H, yellow—Na, wine—Mg.

Initially, to understand the correlation between Mg^2+^ incorporation and precursor concentration, increasing amounts of Mg^2+^ were introduced into the PHI structure by adjusting the initial concentration of the MgCl_2_ solution (Table 1). Its loading was monitored *via* Inductively Coupled Plasma‐Optical Emission Spectroscopy analysis (ICP‐OES, Table S1). In accordance with predictions, the process proceeds in a controlled manner and is governed by the MgCl_2_ solution concentration in which Na‐PHI is suspended. The incorporation curve follows a quadratic trend, where the Mg^2+^ loading rises sharply at lower concentrations until reaching a plateau (Figure S1). In line with this trend, elemental organic analysis revealed a gradual increase in the absolute amount of structural hydrogen. Since thermogravimetric analysis showed no significant variation in the water content (Figure S2), this increase in hydrogen likely indicates a partial protonation process occurring concurrently with the cation‐exchange step. Notably, the highest amount of incorporated Mg^2+^ obtained was around 5.5 wt.%, with a small residual load (2.9 wt.%) of Na^+^ remaining in the structure, indicating that not all the Na^+^ cations can be exchanged by Mg^2+^, even in the more concentrated solution. This ratio corresponds approximately to two Na^+^ for each Mg^2+^ present in the lattice.

**TABLE 1:** Correlation between MgCl_2_·6H_2_O solution concentration and the real loading determined by ICP‐OES.

Label	MgCl_2_ solution concentration (mol L^−1^)
Mg‐PHI 3%	0.05
Mg‐PHI 4%	0.25
Mg‐PHI 4.5%	2.5
Mg‐PHI 5.5%	5.0

To elucidate the structural impacts resulting from the substitution of Na^+^ by Mg^2+^ cations, the material with the highest Mg^2+^ content (Mg‐PHI 5.5%) underwent a comprehensive set of characterization techniques, with the outcoming results being compared to the reference material (Na‐PHI). Initially, the crystalline structure was analyzed using Powder X‐ray Diffraction (PXRD) to identify potential changes in the crystalline lattice and consider the possible formation of oxygenated magnesium species. The periodic arrangement along the plane, indicated by the 100 peak (corresponding to 10.8 Å lattice plane distance), reflects the periodic ordering of symmetrically aligned pores within the in‐plane dimension (Figure 1a) [20]. Additionally, the interlaminar stacking is described by the 001 reflection at approximately 26.3° and 26.8° 2θ for Na‐PHI and Mg‐PHI, respectively, indicating a slight shrinking of the interlayer distance caused by the Na^+^ replacement. As previously reported, the interlayer distance between the sheets is intimately related to the *radius* of the cation residing within the pores, as well as the number of water molecules present in its hydration shell [10]. Since the water content inside the pores did not exhibit significant changes, it is consistent to attribute the sheet contraction to the smaller ionic radius of Mg^2+^ ions [14]. As a typical feature for PHIs synthesized by the bottom‐up approach, the reflections observed between 25° and 27° 2θ are characterized by a series of overlapping and low‐intensity signals, which suggests a randomized packing of the layers [6, 20]. This complex stacking may be attributed to a randomized stacking arrangement of the sheets along the *c*‐direction [21, 31].

**FIGURE 1:**
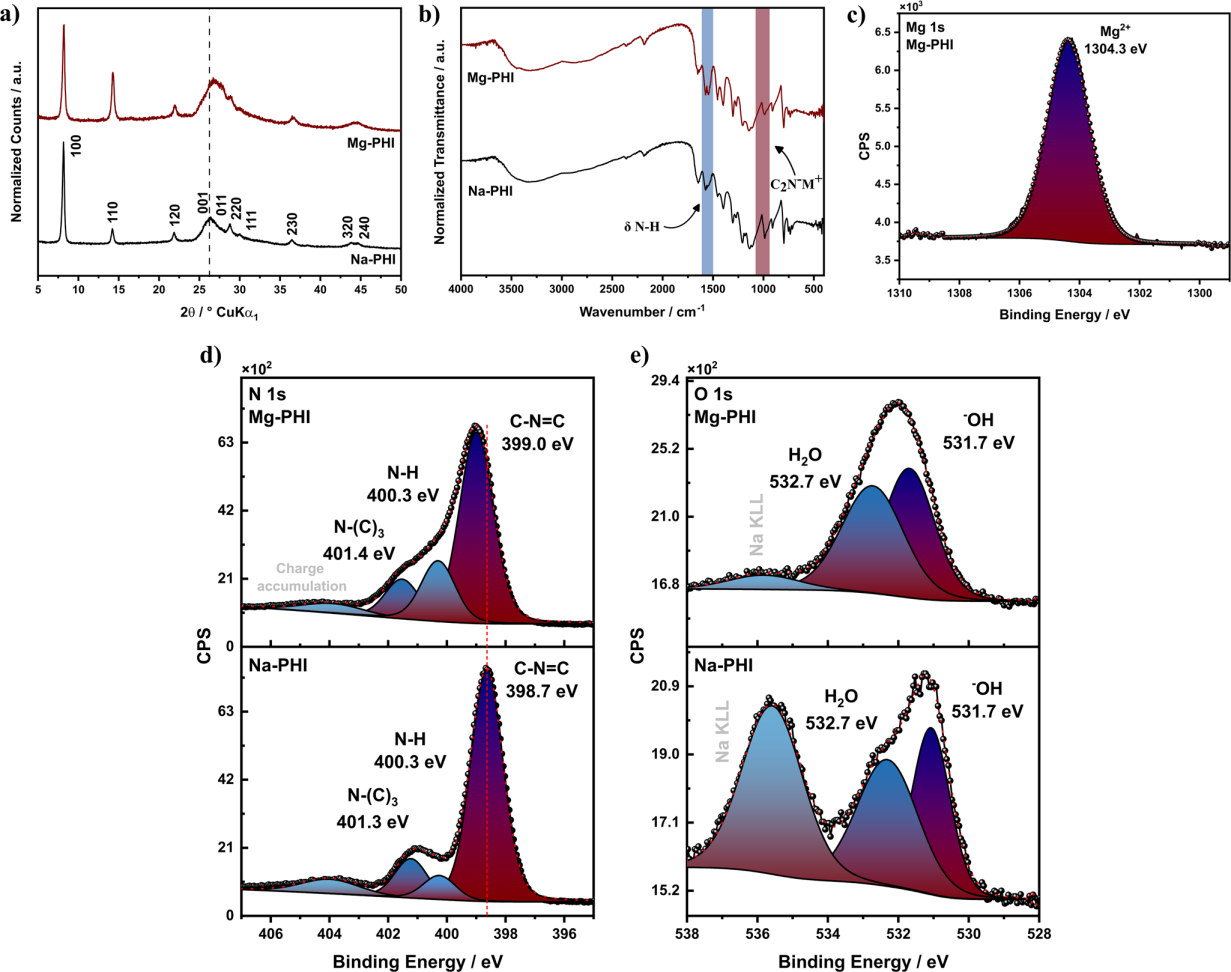
Comparative characterization of Na–PHI and Mg–PHI (5.5 wt.%). (a) PXRD patterns, (b) FT‐IR spectra and (c–e) high‐resolution XPS spectra of the Mg 1s, N 1s, and O 1s regions, respectively.

Essentially, the material exchanged with Mg^2+^ did not exhibit dramatic changes in the crystalline lattice. As demonstrated by a recent study, Na‐PHI and Mg‐PHI are isostructural, discriminated by more rotational disorder for Mg‐PHI [32]. Reflections attributed to MgO and Mg(OH)_2_ are absent in the fresh material (Figure S3a). The final spatial arrangement of the PHI seems to be preserved during the exchange process. Nevertheless, the replacement of Na^+^ by Mg^2+^ resulted in a slight widening of the 100 diffraction plane, while the diffraction peak 110 became more prominent. In principle, changes in signal intensity are expected due to the electron density difference between the cations within the pores. To further investigate the origin of these changes, simplified disordered structural models were constructed by varying only the cation and its corresponding hydration sphere, while preserving the PHI framework. The simulations revealed measurable variations in the interlayer stacking distance (Figure S3b), confirming that the observed diffraction changes originate from cation substitution and its associated hydration environment, rather than from structural modifications of the PHI framework. A deeper understanding of the Mg^2+^ positioning and the chemical environment where these cations are located could provide key insights to unravel their role in the photocatalytic process. However, creating an accurate simulation model for Mg‐PHI remains challenging, as distinguishing between Mg^2+^ species and oxygen atoms from water molecules is impossible from conventional PXRD simulations.

Furthermore, Fourier Transform Infrared spectroscopy (FT‐IR) and X‐ray Photoelectron Spectroscopy (XPS) analyses were performed to verify possible changes in the molecular architecture of the PHI after cation exchange (displayed in Figure 1b–e, respectively). Through the FT‐IR, the distinctive binding framework of Na‐PHI can be highlighted by the typical fingerprint region (1800–1000 cm^−1^), characterized by its heteroaromatic units interlinked *via* negatively charged imide species. The metal presence, interacting with the bridge negative nitrogen centers, is evidenced by the band at 980 cm^−1^ (indicated by the red area). This signal is preserved for Mg‐PHI, confirming the preservation of the ionic character of PHI and thus the presence of cations in the network after the cation exchange. However, the noticeable decrease in intensity suggests partial metal loss. Such feature is commonly observed when metal cations (M^+^) are replaced by protons, converting the electrostatic N^−^‐M^+^ interaction into amine groups. This hypothesis is consistent with the previously discussed increase in hydrogen content within the framework and is further supported by changes in the bending vibration region of N‐H groups (1600–1800 cm^−1^, blue area), where the initially broad doublet undergoes splitting as a result of the cation exchange [33, 34]. Aside from these protonation‐related modifications, the chemical backbone of PHI remains largely unaltered throughout the exchange process. In both spectra, the out‐of‐plane vibration modes of the heptazinic rings are observed at 800 cm^−1^. Moreover, the stretching and vibrational modes associated with C─N and C═N bonds of the conjugated heterocycles are observed in the 1110–1620 cm^−1^ range. The asymmetric stretching of cyano groups (2170 cm^−1^), located at the sheet edges, appears as a weak signal, associated with incomplete polymerization of the heptazine framework [35, 36]. Finally, bands related to adsorbed water and terminal amine groups are visualized and located at 3000–3500 cm^−1^. For those last‐mentioned bands, no changes were observed.

Regarding the XPS analysis, the high‐resolution regions corresponding to O 1s, N 1s, and Mg 1s (Figure 1c–e) were examined. In the Mg 1s high‐resolution window (Figure 1c), a single peak at 1304.3 eV (typical for Mg^2+^) is observed, confirming its presence in the composition of the final material [37, 38]. The N 1s region (Figure 1d) basically displays the characteristic signals associated with PHI‐type structures [30, 39]. When comparing the N 1s spectra of Na‐PHI and Mg‐PHI, the Mg‐PHI sample exhibits a higher contribution from ‐NH nitrogen species at ∼400.3 eV, suggesting partial surface protonation at these sites upon Mg incorporation, which is in agreement with the FT‐IR observations. In addition, the peak associated with C─N═C nitrogen appears at ∼399.0 eV for Mg‐PHI, showing a positive shift of approximately 0.3 eV relative to Na‐PHI. This shift suggests a decrease in electron density within the heptazine framework upon Mg^2+^ incorporation, which can be attributed to the higher valence of Mg^2+^ compared to Na^+^, leading to a stronger electrostatic interaction with the nitrogen sites. In the O 1s region (Figure 1e), both spectra exhibit contributions from surface hydroxyl groups (∼531.7 eV) and physisorbed water molecules (∼532.7 eV), with no evidence of oxygenated Mg species [37, 40].

From an electronic standpoint, incorporation of Mg^2+^ does not induce significant changes in the optical absorption edge of the material, which remains centered at approximately 2.74 eV (Figure 2a). Consistently, steady‐state photoluminescence spectra display broad emission bands centered around 470 nm for all samples. Previous studies on carbon nitride‐based materials, such as melon, have demonstrated complex recombination pathways attributed to the heterogeneity of the polymer. To evaluate potential changes in the band positions of PHI upon Mg^2+^ incorporation, frequency‐dependent Mott–Schottky analysis (Figure 2b,c) was employed to determine the conduction band position, while XPS measurements in the core‐level region (Figure S4c,d) were used to estimate the valence band maximum. As shown in Figure 2d, both the valence and conduction band edges exhibit downward shifts upon Mg^2+^ incorporation, rendering the material more reducing.

**FIGURE 2:**
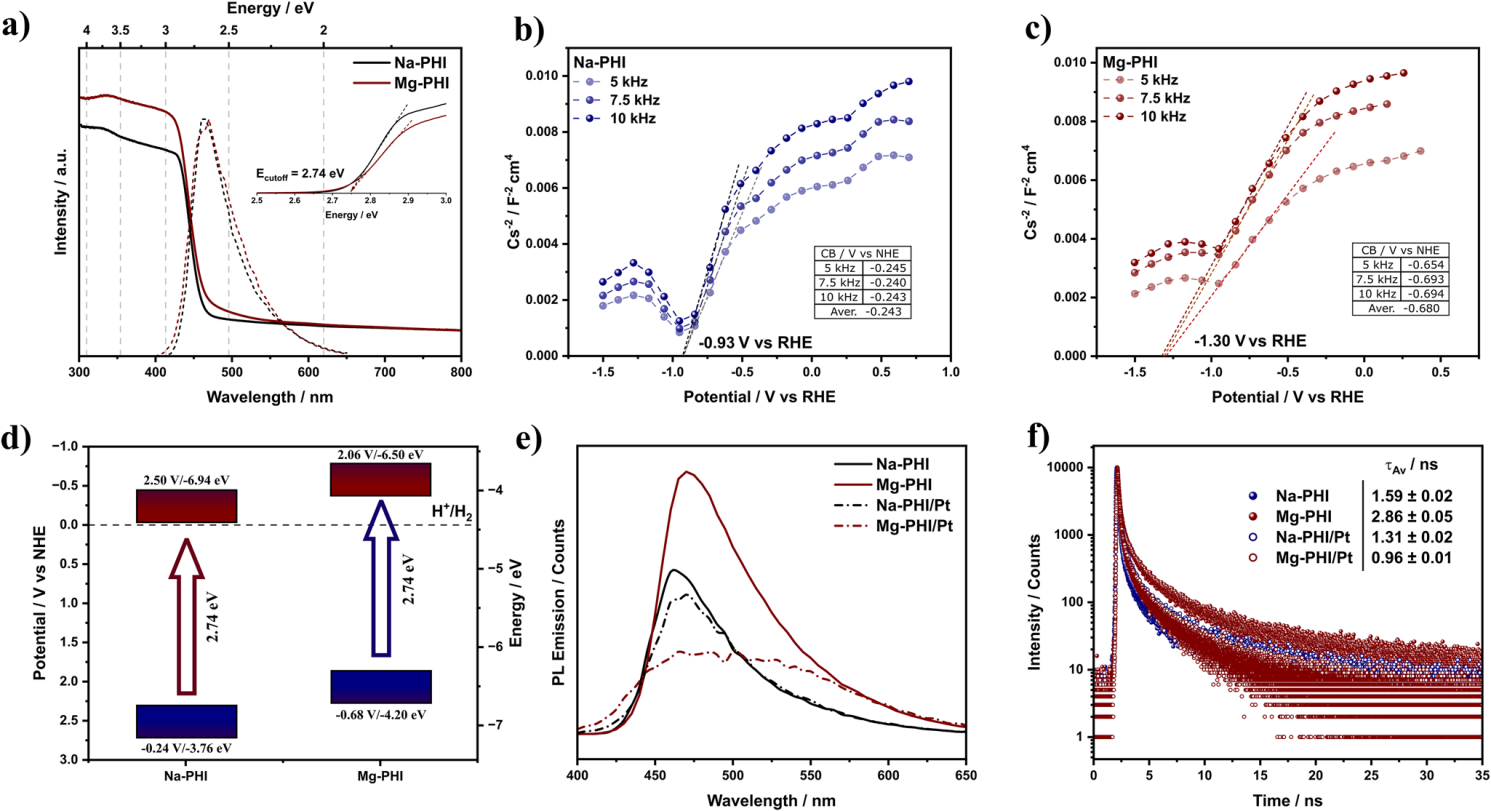
Optoelectronic properties of Na–PHI and Mg–PHI (5.5 wt.%). (a) Diffuse reflectance spectra corresponding to the solid lines and emission signatures by the dashed lines; the inset displays the Tauc plots assuming an indirect transition, following the method proposed by Makula et al. [41]; (b,c) Frequency dependent Mott–Schottky plot for the flat‐band potential determination according to the method proposed by Hankin et al. [42]; (d) band structure as a function of potential vs. Normal Hydrogen Electrode (NHE), determined from Mott–Schottky analysis accompanied by the corresponding energy scale shown on the right‐hand axis based on valence band maxima determined by XPS. In situ photoluminescence plot for both materials and their Pt‐containing counterparts: (e) steady‐state emission spectra and (f) time‐resolved photoluminescence decay curves in the logarithm scale, including the calculated average lifetimes.

In addition, we investigated the photophysical relaxation pathways through in situ photoluminescence (PL) measurements, analyzing the materials in the absence and presence of Pt. At first, it is noteworthy that Pt‐containing samples exhibited significantly reduced photoluminescence intensity, which is expected based on the Pt role as electron‐trapping site, preventing exciton recombination. However, a subtle quenching decrease in emission intensity was observed in Na‐PHI/Pt, which was markedly enhanced in the Mg‐PHI/Pt system (Figure 2e). This observation suggests that Mg^2+^ plays a critical role in facilitating more effective electron transfer toward Pt sites.

This hypothesis is further supported by in situ time‐resolved photoluminescence (TR‐PL) measurements (Figure 2f). Remarkably, substituting Na^+^ with Mg^2+^ in the absence of Pt significantly increased the average carrier lifetime from approximately 1.6 to 2.8 ns. However, upon Pt deposition, the average lifetime notably decreased in both systems, from 1.6 to 1.3 ns in Na‐PHI and more markedly in the Mg‐PHI, from 2.8 to 0.9 ns, in line with the steady‐state PL observations.

It is important to emphasize that these measurements were conducted simulating the reaction environment, i.e., in the presence of triethanolamine (TEOA) and under oxygen‐free conditions. As a consequence, photogenerated holes are efficiently quenched, and the plausible pathways for photogenerated electrons are limited to either recombination or reduction of water. Given that PHI is an intrinsically poor electron donor, electron transfer toward water is inefficient in the absence of Pt, as no suitable catalytic sites are available. In contrast, upon Pt deposition, proton reduction becomes kinetically accessible, leading to a pronounced decrease in carrier lifetime. In this regime, the more substantial lifetime shortening observed for Mg‐PHI is particularly noteworthy. Since no structural changes are detected upon Mg^2+^ incorporation, this behavior can be ascribed to more efficient charge transport in Mg‐PHI compared to Na‐PHI.

Detailed analysis of fitting parameters from TR‐PL decay curves offers insights into how Mg^2+^ incorporation impacts the recombination dynamics (see Figures S5 and S6). While PHI structures have recently gained attention in the carbon nitride community, an in‐depth understanding of their photorelaxation mechanisms remains limited. Nevertheless, again, analogies can be drawn from the most extensively studied melon, which shares structural and compositional similarities with PHI. In melon, it is well established that electronic motion mainly occurs *via* interlayer migration, mediated through π–π interactions between aligned aromatic orbitals [43]. Thus, the decay components obtained in the current systems (Tables S4–S7) indicate that in Na‐PHI, Pt deposition predominantly reduces the lifetime of carriers involved in fast recombination events, related precisely to interlayer recombination events (τ_3_), while longer‐lived components, associated with short‐range intrachain migration (τ_2_) and electrons confined within the heptazine orbitals (τ_1_), remain largely unaffected [44]. This suggests that, in Na‐PHI/Pt, Pt effectively intercepts electrons travelling perpendicular to the plane of the sheets near their excited states, whereas electrons involved in longer‐range migration or trapped states remain spatially or energetically decoupled from Pt. Conversely, the Mg‐PHI/Pt system displays significant lifetime reductions across all components (τ_1_, τ_2_ and τ_3_) upon Pt deposition. This comprehensive quenching behavior implies that Pt‐mediated charge extraction in Mg‐PHI extends beyond localized excitation sites, encompassing both short and long‐range migrating carriers.

### 2.2 Photocatalytic H_2_ Evolution

Since structural analysis confirmed that the cation exchange preserves the material's structure, the electronic influence of the alkaline‐earth metal on photocatalytic efficiency can now be isolated, therefore enabling access to the direct impacts of the presence of Mg^2+^ in the network. Accordingly, we compared the hydrogen evolution rates of Mg‐PHI against Na‐PHI to quantify the promoting effect of magnesium present within the framework. The photocatalytic tests were performed under 410 nm LED (68.1 mW cm^−2^) irradiation and in a photoreactor containing a solution (deionized water/TEOA 10%) and 25 mg of the photocatalyst.

We first investigated the effect of cation concentration by evaluating a series of materials with increasing Mg content (Figure 3a). Notably, a positive linear correlation was observed between metal loading and photocatalytic performance. The most active sample, Mg‐PHI 5.5%, was able to reach a hydrogen production rate of 18909 µmol g^−1^ h^−1^ and 9.19% of Apparent Quantum Yield (AQY), corresponding to a 2.5‐fold enhancement compared to Na‐PHI. Given the pronounced influence of magnesium incorporation, the system behavior was further examined by varying the amount of photodeposited Pt on optimized Mg‐PHI, using Na‐PHI as a reference. As shown in Figure 3c, Mg‐PHI consistently exhibited superior hydrogen evolution activity compared to Na‐PHI across all platinum loadings. Notably, for Na‐PHI, increasing the amount of photodeposited platinum had little to no effect on the H_2_ production rate, with the reaction rate rapidly reaching a plateau even at low Pt loadings, indicating that co‐catalyst availability is not the rate‐limiting factor in this system. Conversely, Mg‐containing samples showed a linear dependence on Pt availability, achieving a peak activity of 20581 µmol g^−1^ h^−1^ (Mg‐PHI 5.5%, 6 wt.% Pt). Importantly, this linear kinetic response suggests that the activity is governed by interfacial electron‐transfer processes, which become progressively more efficient as Pt sites increase. Since this enhancement is observed only when both the alkaline‐earth metal and the Pt co‐catalyst are present, the results suggest a synergistic interaction consistent with the formation of interfacial charge‐bridging pathways.

**FIGURE 3:**
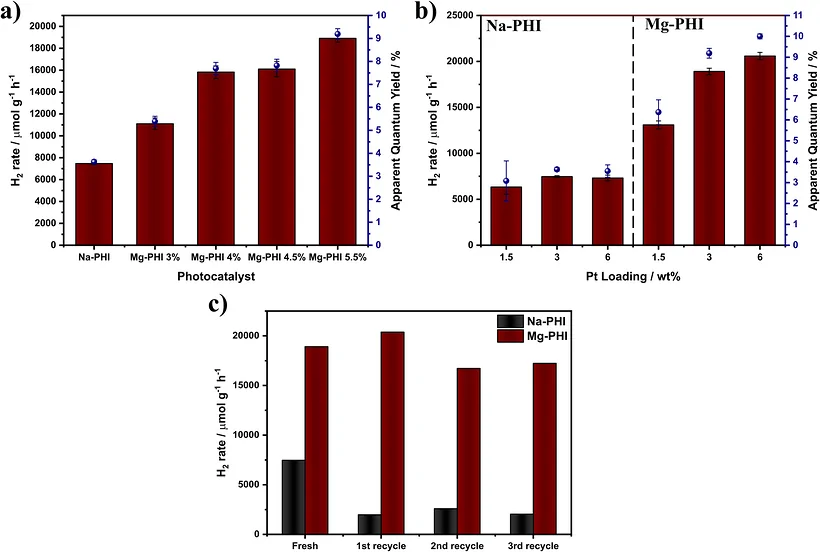
Photocatalytic hydrogen evolution performance under visible‐light irradiation (410 nm LED, 68.1 mW cm^−2^). (a) Dependence of H_2_ evolution rate on Mg^2+^ loading in PHI using a fixed Pt co‐catalyst content (3 wt.%); (b) comparison of the influence of photodeposited Pt loading on Na‐PHI and Mg‐PHI (5.5%), highlighting a pronounced enhancement for Mg‐containing samples; (c) Recycling tests.

It is worth noting that this synergistic behavior was also observed under both photo‐ and electrochemical conditions, as discussed in detail in the Supporting Information. Control experiments were performed to independently assess the contributions of Mg^2+^ and Pt to photocurrent generation, electrical resistance, and charge generation, separation, and transport (Figures S7 and S8). Overall, the (photo)electrochemical results are fully consistent with the photocatalytic data, demonstrating that the presence of Mg^2+^ within the PHI framework significantly enhances conductivity and charge‐transfer efficiency, which can be associated to the bivalent nature of Mg^2+^ cations, as established by Kröger et al. [10]. Furthermore, our results underscore that this effect becomes more pronounced in the concomitant presence of Pt, highlighting the modulation of the charge dynamics dominance by the metal co‐catalyst, either by generating a local electric field that facilitates charge separation and transport, or by acting as trap states that stabilize photogenerated charges [45].

Subsequently, the photocatalysts were subjected to stability tests to evaluate their behavior over successive reaction cycles. After each photocatalytic run, the materials were recovered by centrifugation, extensively washed with deionized (DI) water to remove adsorbed species, especially residual TEOA and its oxidation products, and then reused in consecutive tests without additional Pt deposition. As an initial observation, Na‐PHI exhibited a loss of approximately half of its original activity, a performance that persisted up to the third recycling cycle. In contrast, Mg‐PHI demonstrated good stability even after the third recycling test. Previous studies have shown that PHI materials, specially Na‐PHI, are susceptible to structural protonation under photocatalytic hydrogen evolution conditions, i.e., replacement of the pore‐compensating cations by protons, thus leading to catalyst deactivation despite the mildly alkaline reaction medium (pH 10.5–11.0) [46]. Consistent with the literature, the FT‐IR spectrum of the recycled Na‐PHI sample exhibits the appearance of a new band centered at approximately 1550 cm^−1^, associated to N─H bonds, indicating structural protonation (Figure S17a). In contrast, Mg‐PHI displays only a minor decrease in photocatalytic activity after repeated reaction cycles.

No significant changes were detected in the crystalline structure of the PHI framework after the reaction (Figure S17b). However, additional broad reflections attributable to Mg(OH)_2_ were observed for the post‐reaction Mg‐PHI sample. Likewise, no substantial changes were identified in the optical absorption spectra of either material (Figure S17c). XPS analysis of the post‐reaction samples (Figure S17d–f) revealed a clear shift of the Mg 1s peak toward lower binding energies, indicating an increase in the electron density around the Mg^2+^ coordination environment after catalysis. A similar trend was observed for the C─N═C nitrogen species, whose binding energy shifted back toward values comparable to those of Na‐PHI. These changes are consistent with a weakening of the interaction between the PHI framework and Mg^2+^ cations, possibly arising from the coordination of hydroxide species generated during the reaction.

Quantitative XPS analysis indicates that the surface Mg^2+^ content remains essentially unchanged throughout the reaction (Tables S3 and S12). However, by ICP we detected a reduction in the overall loading of Mg^2+^ to 1.2 wt.%. Taken together, this observation suggests that Mg loss occurs predominantly from the bulk of the material, while the surface Mg^2+^ species, presumably responsible for the catalytic function, remain largely preserved.

TEM analysis of the post‐reaction materials was performed to evaluate whether the nature of the cation influences the spatial distribution of the photodeposited Pt co‐catalyst (Figures S14 and S16). The images reveal a homogeneous dispersion of Pt nanoparticles and Mg species across the PHI matrix, with no evidence of large segregated domains. In both systems, Pt nanoparticles with average size in the 45–50 nm range were observed, indicating that Mg incorporation does not significantly affect the Pt photodeposition process. Interestingly, EDS elemental maps of Mg‐PHI 5.5% after catalysis (Figure S16) show a homogeneous distribution of both Mg and O throughout the material, with no visible agglomerates associated with the formation of large magnesium hydroxide crystallites.

To directly assess whether Mg(OH)_2_ contributes to the photocatalytic response, control experiments were performed using Na‐PHI impregnated with Mg(OH)_2_ by dry impregnation. Notably, this material exhibited no detectable H_2_ production and exhibited inferior performance compared with pristine Na‐PHI, ruling out Mg(OH)_2_ as the catalytically active phase. Instead, these results further support that Mg^2+^ in its cationic form governs the interfacial interaction responsible for the enhanced photocatalytic activity.

Owing to the strong polarizing ability of Mg^2+^ cations and their synergistic interaction with the Pt co‐catalyst, as indicated by the hydrogen evolution measurements, the formation of a solid‐state Mg^2+^‐OH^δ−^‐H^δ+^‐Pt interfacial motif is proposed. Within this interface, Mg^2+^ acts as a catalytic promoter by kinetically facilitating water heterolysis and the subsequent transfer of protons to the Pt nanoparticles, where they are reduced to H_2_. Post‐reaction characterization further indicates that the hydroxide species concomitantly generated during this process remain coordinated within the Mg^2+^ coordination sphere.

### 2.3 Computational Simulations

The formation of the Mg^2+^‐OH^δ−^‐H^δ+^‐Pt interface hypothesis was then analyzed by computational simulation evaluating the local distribution of electronic density in the vicinity of the Pt co‐catalyst. For this purpose, a molecular dynamics (MD) model was constructed to represent Mg‐PHI 5.5% containing the co‐catalyst, based on experimental data derived from the crystalline architecture, elemental composition and optical absorption. The simulated UV–vis spectrum accurately reproduces the key features of the experimental spectrum (Figure 4a). The natural transition orbital (NTO) with the highest oscillator strength around the wavelength of 410 nm (experimental region of interest) was analyzed. The average NTO iso‐surfaces at 99% probability of finding hole and electron (Figure 4b) show a high degree of delocalization for both the hole and the electron across the PHI stack, which is consistent with a structure governed by π − π stacking interactions. Reducing probability to 90% provides a more localized representation of the hole and electron iso‐surfaces (Figure 4c), allowing to identify the most favorable regions for hydrogen production, which must correspond to regions close to water molecules where the hole density is negligible and the electron density is larger. The two left‐hand insets highlight two regions where this occurs, in which the electron iso‐surface localized near the water molecules surrounding the Mg^2+^ cations in two pore channels adjacent to the nanoparticle. To investigate the Pt nanoparticles and the PHI contribution in other transitions, we searched for over a total of 2500 averaging NTOs, in an energy range from 0.055 to 3.094 eV. Our analysis revealed that the most significant Pt contributions are concentrated in the NIR region, as might be expected for metallic materials, and the largest Pt contribution for the hole NTOs was only 4.89%. Therefore, our excited‐state simulations reveal that Pt atomic orbitals contribute only marginally to the initial photoexcited states, indicating that the Pt nanoparticle does not directly participate in the formation of the primary excited state. The primary photoexcitation is mainly localized within the PHI framework and the surrounding adsorbed molecules.

**FIGURE 4:**
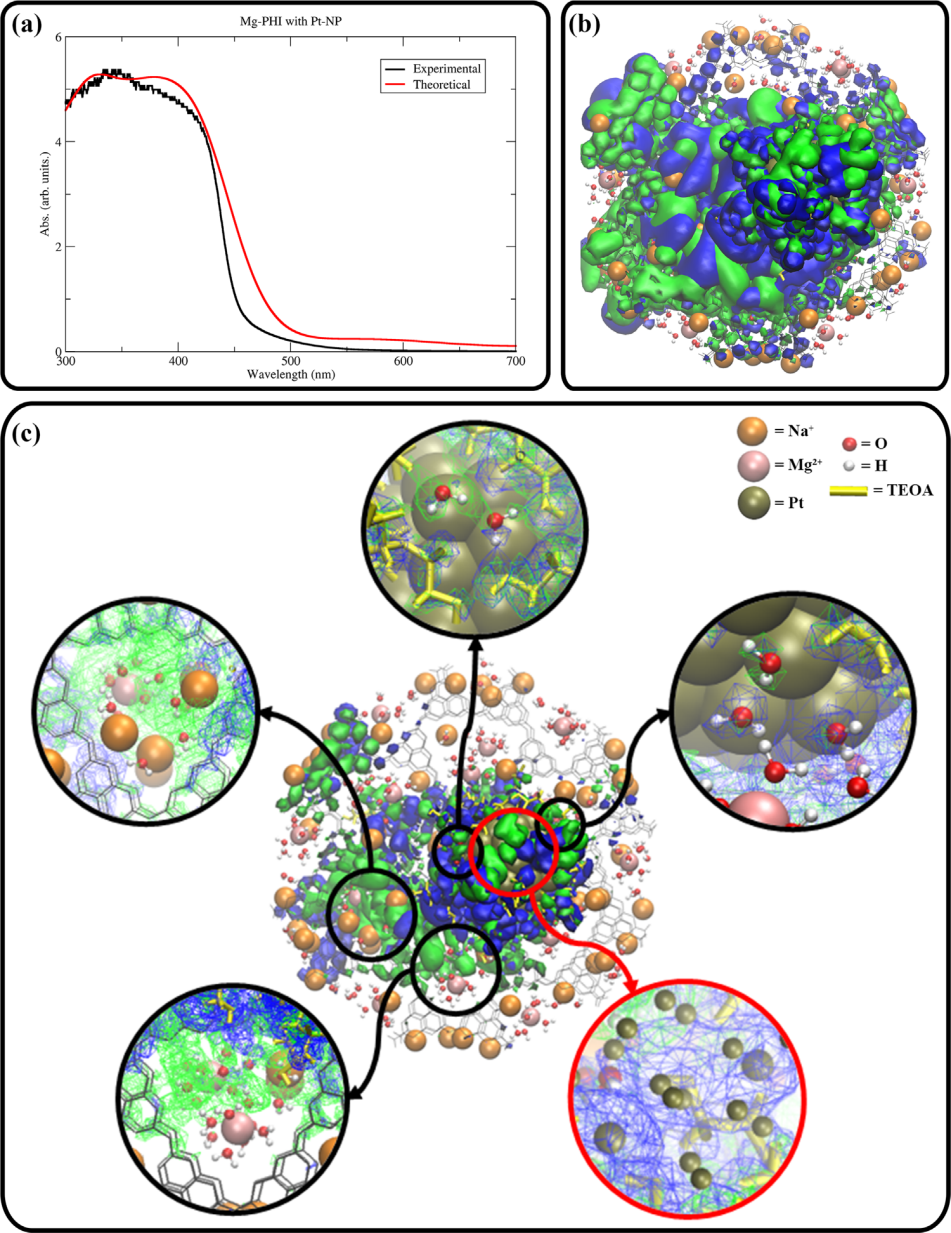
(a) Experimental and simulated UV–vis spectrum. The oscillator strengths of the simulated spectrum were convoluted using a Gaussian broadening with full width at half maximum of 0.2 eV (the simulated spectrum was blue‐shifted by 50 nm to improve the matching with the experimental curve). Averaged NTO calculated for the transition with the highest oscillator strength in the spectral window close to the experimental irradiation wavelength of 410 nm, corresponding to the transition at 407.8 nm, shown. The hole and electron orbitals are depicted as blue and green iso‐surfaces, respectively, with probabilities of 0.99 in (b) and 0.90 in (c), with insets showing details of some interesting regions. In each inset, the isosurfaces are shown in a more transparent way for a better visualization.

The nature of the excited state plays a decisive role in the photocatalytic mechanism. Limited spatial separation between the hole and electron favors rapid recombination, whereas greater charge separation can extend the excited‐state lifetime and enable electron transfer to reducible species. Because the hole density within these channels is negligible, the corresponding excitations exhibit pronounced charge‐transfer character. In contrast, within the PHI sheets and TEOA molecules, the hole and electron densities remain spatially close, indicating a predominantly locally excited character. The subsequent transitions with the highest oscillator strengths in the same spectral region were characterized either by substantial electron–hole overlap, and thus a high recombination probability, or by electron density localized far from the water molecules (Figure S19).

The upper inset of Figure 4c highlights two isolated water molecules located near the Pt nanoparticle and involved in a charge‐transfer excitation. However, the close spatial proximity of the hole and electron isosurfaces favors rapid recombination, making this configuration unlikely to contribute substantially to the photocatalytic process. Nevertheless, the presence of electron density on the water molecules indicates that direct electron transfer to adsorbed water is electronically accessible in this system. By contrast, in the configurations shown in the left‐hand insets, where Mg^2+^ cations are located near the Pt nanoparticle, a markedly greater spatial separation of the charge carriers is observed, with electron density predominantly accumulated around the Mg‐coordinated water molecules in the interfacial region. This result demonstrates that the local proximity between Mg^2+^ and the Pt co‐catalyst is critical for promoting such charge redistribution, consistent with the stronger photoluminescence quenching observed for samples containing both Mg and Pt.

The hole isosurfaces are predominantly localized on the sacrificial TEOA molecules adjacent to the nanoparticle and within the PHI sheets. Pt atomic orbitals contribute only marginally to the excited states examined. Although the lower‐right inset of Figure 4c shows a small fraction of hole density localized on two specific Pt atoms, represented by larger spheres, this contribution remains minor. Nevertheless, it may indicate a secondary electron‐transfer pathway from TEOA to Pt, raising the possibility that Pt plays a dual interfacial role by promoting proton reduction while also mediating TEOA oxidation.

### 2.4 In Situ XANES Essays

Experimentally, the proposed interaction was validated by in situ X‐ray Absorption Near Edge Structure (XANES) measurements, in which changes in the electronic density of Mg^2+^ were probed under simulated photocatalytic operating conditions identical to those employed in the hydrogen evolution experiments (Figure 5). Both Na‐ and Mg‐based PHI materials were compared in the presence and absence of Pt. As an initial reference, the influence of water on the metal geometry and electronic structure was evaluated by measuring the fully dried samples, previously dehydrated at 250°C (dark‐blue lines). Subsequently, the materials were photoexcited using a purple laser (408.63 nm, 40 mW), while a continuous water flow was simultaneously introduced into the system (light‐blue lines). Under these conditions, changes in the electronic density and local symmetry of the metal centers could be directly monitored, arising from photoinduced electron transfer followed by proton reduction. To further disentangle the individual contributions of light and water, an additional spectrum was collected after interrupting the water flow while maintaining laser irradiation. Finally, a last measurement was acquired after switching off the laser, enabling direct assessment of the reversibility of the electronic changes observed during photocatalytic operation.

**FIGURE 5:**
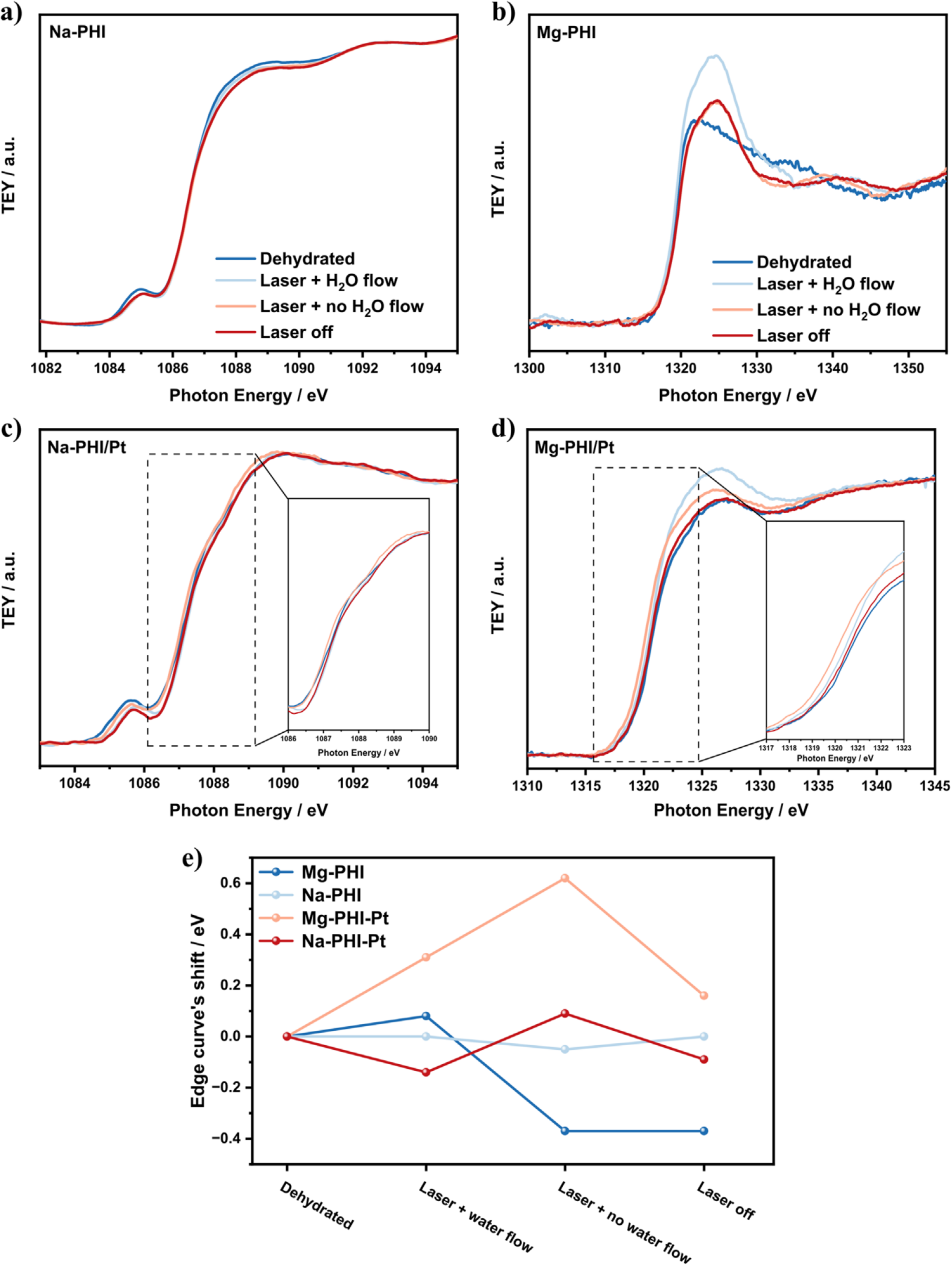
In situ XANES of the K edge spectra of: (a,c) Na and (b,d) Mg metals of the photocatalysts without (upper panels) and with (lower panels) Pt co‐catalyst under simulated reaction conditions. (e) The respective shift of the K edge curve plotted according to each analysis condition. TEY: Total Electron Yield.

Initially, reference measurements were taken for the materials without the Pt co‐catalyst to evaluate the natural response of the studied metal edges under the given conditions. At the Na K‐edge (Figure 5a), illumination of the sample in flowing H_2_O causes a small decrease of the pre‐edge peak at ca. 1085 eV, which is in turn due to Na 1*s*→3*s* transition [47]. Although this transition is dipole‐forbidden, it can become weakly allowed if the local symmetry of Na is not strictly centro‐symmetric. A decrease in intensity of this feature can be attributed to an increase of the occupation of Na 3s orbitals and/or to a more ordered local chemical environment around the Na sites, which increases the symmetry. Both these effects can be related to the adsorption of water on the Na sites. It is important to note that these changes in intensity are not reversed, neither by switching the laser off, nor by interrupting the water flow, suggesting that H_2_O adsorption is strong and can only be removed totally by heating. Other minor changes are detected at ca. 1088 eV, where the dipole allowed Na 1*s*→3*p* transitions are found [47]. Also in this case, a largely non‐reversible decrease in intensity is found, which can be likewise attributed to water adsorption, resulting in an increase of the occupation of Na 3*p* states. For the Mg K‐edge (Figure 5b), a marked increase in intensity of the main edge peak is found by flowing water under illumination, which can be partially reversed by stopping the H_2_O flow. Again, this variation in intensity is attributed to water adsorption, in agreement with previous literature [48]. The larger changes compared to the Na‐containing material reveal that coordination of water around Mg cations has a greater impact on the local electronic density compared to Na.

For Na‐PHI containing platinum co‐catalyst (Figure 5c), two distinct features are detected at the Na K‐edge: a decrease in intensity of the pre‐edge peak as discussed above, and a shift of the edge absorption energy to a lower energy (by 0.15 eV, as better evidenced in the inset). For the material containing Mg in the presence of platinum (Figure 5d), an increase in intensity of the main edge peak is again detected, but to a lower extent than in the previous case. Additionally, a shift of the edge energy position toward lower energy is revealed under illumination and in flowing water. As evidenced by the inset, which shows the edge energy region on an enlarged scale, such shift amounts to 0.57 eV, almost four times larger than for sodium. After exposing the samples to these conditions, the water flow was interrupted, and the laser was switched off. Thereby, it was possible to observe the progressive return of the edge to its initial position, the most marked shift being produced by stopping the illumination, after having stopped the water flow.

We note that the edge energy position is a measure of the binding energy of the 1*s* electrons. This is due to the Coulomb interaction between such electrons and the nucleus, and it is in turn modulated by all the outer (valence) electrons. The larger the shielding the lower the binding energy and, therefore, the lower the edge energy position. Thus, a shift of the edge toward lower energies is a clear indication of an increase in the electron density around Na and Mg, which is revealing a decrease of the charge state. This effect is significantly larger for Mg than for Na and it is detected only in the presence of Pt (Figure 5e).

In summary, by integrating all the obtained results, a mechanism for the hydrogen evolution reaction is proposed in Figure 6. Upon photoexcitation, photogenerated charges migrate and accumulate on the Pt co‐catalyst. Simultaneously, water molecules located within the coordination sphere of Mg^2+^ and sufficiently close to Pt sites become polarized, thereby facilitating proton transfer to the metal surface. The transferred protons are subsequently reduced to H_2_ at the Pt sites, while the corresponding hydroxide species are stabilized in the coordination sphere of Mg^2+^. In addition, Na^+^ and Mg^2+^ act as preferential sites for water adsorption, and a cooperative interaction emerges between Na^+^/Mg^2+^ centers within the PHI framework and Pt nanoparticles acting as co‐catalysts, with the synergistic effect being most pronounced in Mg‐containing samples.

**FIGURE 6:**
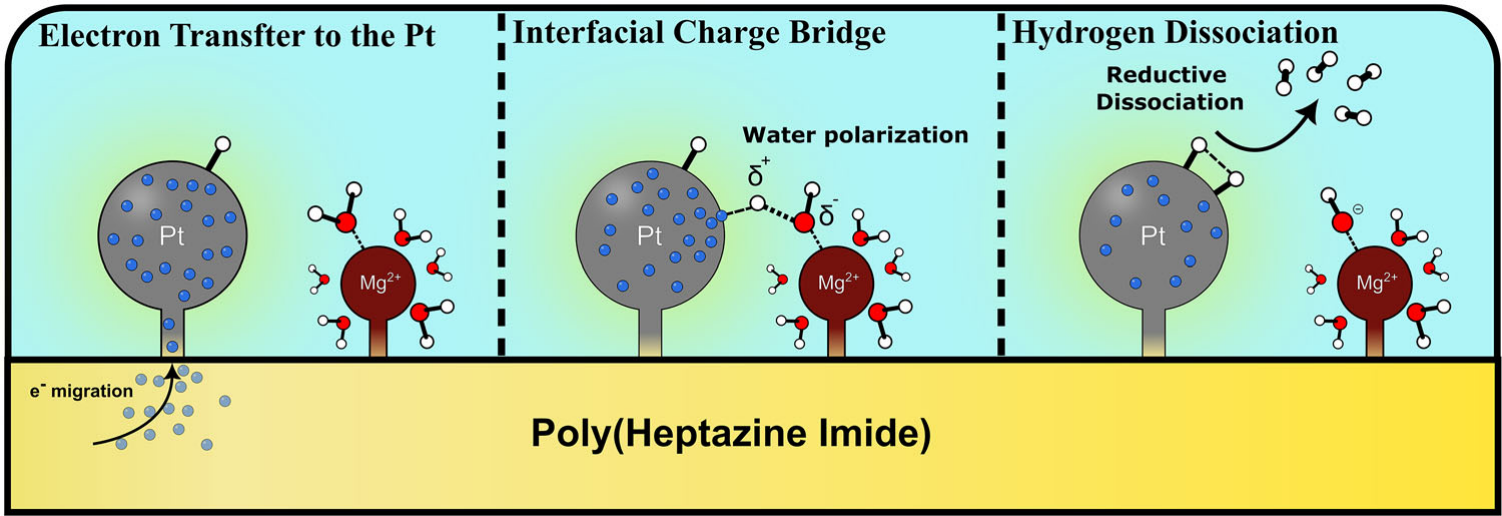
Schematic illustration of the proposed mechanism highlighting the cooperative role of Mg^2+^ centers in polarizing water molecules within their coordination sphere, thereby facilitating proton transfer to the Pt surface and promoting efficient photocatalytic hydrogen evolution.

## 3 Conclusions

This work unravels the mechanistic origins of cation‐promoted photocatalysis, identifying the pivotal role of Mg^2+^ in enhancing hydrogen evolution over PHI matrices. By integrating experiments and theory, we demonstrated that Mg^2+^ is not merely a structural spectator but acts as an active charge‐transfer bridge between the polymeric support and the Pt co‐catalyst. Our data reveal that this synergy relies heavily on spatial proximity: in situ XANES and molecular dynamics simulations confirm an electronic density enrichment at the Mg^2+^‐OH^δ−^‐H^δ+^‐Pt interface. Under operating conditions, this interaction polarizes water molecules within the Mg^2+^ coordination sphere, facilitating proton transfer and stabilizing intermediate hydroxide species. This orchestrated mechanism resulted in a 2.5‐fold enhancement in catalytic activity. Most significantly, the principles established here extend beyond hydrogen evolution. The “cation‐mediated bridging” mechanism is generic and relies solely on the spatial co‐presence of the cation and the metal co‐catalyst. Therefore, this strategy offers a universal design principle applicable to a broad spectrum of reduction chemistries, from CO_2_ activation to complex organic transformations, paving the way for the rational design of next‐generation hybrid photocatalysts.

## 4 Experimental Section/Methods

### 4.1 Characterization

#### 4.1.1 Powder X‐Ray Diffraction

PXRD experiments were performed utilizing a Stadi P device provided by Stoe equipped with a Ge(111) monochromator. The samples were sealed in a 0.7 mm capillary tube and analyzed using a pure Cu‐Kα1 (λ = 1.5406 Å) radiation source and employing the Debye‐Scherrer geometry acquisition mode. The covering range was collected from 5 to 50° with a step size of 0.015° and counting time of 140 s step^−1^.

#### 4.1.2 Fourier Transform Infrared Spectroscopy

FT‐IR analysis were conducted using a Spectrum Two spectrophotometer provided by Perkin Elmer coupled with an Attenuated Total Reflectance (ATR) module featuring a diamond crystal. The spectral acquisition ranged from 400 to 4000 cm^−1^, with a spectral resolution of 4 cm^−1^ and accumulation of 25 scans.

#### 4.1.3 UV–Vis Diffuse Reflectance Spectroscopy

DRS measurements were carried out using a UV‐2600i spectrophotometer purchased from Shimadzu. The data were collected using reflectance operation configuration scanning in the range of 200–900 nm with 0.1 nm of spectral resolution.

#### 4.1.4 X‐Ray Photoelectron Spectroscopy

XPS was performed using a Kratos Axis‐Ultra system equipped with a monochromator and an Al Kα source (hν = 1486.6 eV). Survey scans were acquired with a pass energy of 80 eV while high‐resolution spectra were measured with a pass energy of 20 eV. A charge neutralizer was used to compensate for surface charging and all spectra obtained were calibrated using the binding energy of the adventitious carbon bond (C─C), set at 284.8 eV [49]. CasaXPS was used for data analysis. Fitting was performed by using a convolution of Gaussian and Lorentzian line shape after subtraction of a Shirley background. Quantification was done from the Mg 1s spectra using sensitivity factors provided by the instrument manufacturer and implemented in CasaXPS [50].

#### 4.1.5 Transmission Electron Microscopy

TEM imaging was conducted using a JEOL image‐corrected ARM200F microscope equipped with a FEG gun operating at 200 kV.

#### 4.1.6 Photoluminescence and Time‐Resolved Photoluminescence Spectroscopy

PL and TR‐PL spectra were acquired using a FluoTime 300 spectrometer (PicoQuant). The analysis was performed in suspension mimicking the reactional conditions (in situ); thus 2 mg of sample was suspended in 2 mL of a solution water‐triethanolamine (10% v/v) previously degassed. The procedure was done using a glovebox to avoid molecular oxygen in the medium. Initially, the steady‐state spectrum was recorded using a 375 nm excitation laser to determine the maximum emission wavelength. For time‐resolved measurements, the monochromator was fixed at 470 nm (wavelength with maximum emission). The Instrument Response Function (IRF) was previously acquired using a LUDOX suspension as scattering standard. The decay fitting was performed using a triple‐exponential (*n_Exp_* = 3) decay model, according to the following equation:

$$\begin{array}{ccc} \mathrm{Dec}\left(t\right) & = & \left[\int_{-\infty }^{t}dt^{\prime }\left[IRF\left(t-Shift_{IRF}\right)-Bkgr_{IRF}\right]\right]\left[\sum_{i=1}^{n_{Exp}}A_{i}e^{-\frac{t-t^{\prime }}{\tau_{i}}}\right]\\ & + & Bkgr_{Dec} \end{array}$$

$$\tau_{Av}=\sum_{\begin{array}{c} i=1\\ A_{i}>0 \end{array}}^{n_{Exp}}A_{i}\tau_{i}/\sum_{\begin{array}{c} i=1\\ A_{i}>0 \end{array}}^{n_{Exp}}\tau_{i}$$

where τ_1_​, τ_2_​, and τ_3_ represent the decay lifetimes and A_1_​, A​_2_, and A_3_​ are the corresponding amplitude coefficients.

#### 4.1.7 Thermogravimetric Analysis

TGA‐DTG measurements were carried out in a STA 449 F3 Jupiter device fabricated by Netzsch. The samples were analyzed under different Ar and synthetic air atmospheres heating from room temperature up to 800°C with a heating rate of 10°C min^−1^.

#### 4.1.8 Elemental Analysis

Inductively Coupled Plasma—Optical Emission Spectroscopy (ICP‐OES) and organic analysis (CHN) data were obtained using a Varian Vista‐PRO and UNICUBE elemental analyzer system. The samples were dissolved using a microwave digestion with SP‐D device from CEM GmbH in HNO_3_ at 185°C for 25 min and then diluted with deionized water.

#### 4.1.9 Mott–Schottky Curves

The Mott–Schottky analysis were recorded with potential range from 0.7 to −1.5 V (−0.1 V step) vs. Reversible Hydrogen Electrode (RHE), a potential amplitude of 10 mV, frequencies 5, 7.5, and 10 kHz, and the electrolyte used was Na_2_SO_4_ 0.5 mol L^−1^. The conversion from RHE to Normal Hydrogen Electrode (NHE), the Nernst equation was employed.

#### 4.1.10 Electrochemical Measurements

Linear sweep voltammetry (LSV), chronoamperometry (CA) and electrochemical impedance spectroscopy (EIS) measurements were recorded in a three‐electrode system using a PGSTAT 204 potentiostat, from Metrohm Autolab, interfaced to a computer controlled by NOVA software version 2.1.5. To prepare the FTO/M‐PHI working photoelectrodes (1 cm^2^), the synthesized material was initially dispersed in a solution containing 1.5 wt.% polyvinylidene fluoride in 1‐methyl‐2‐pyrrolidone, in a 9:1 ratio, respectively, and placed to an ultrasonic bath for 24 h [51]. After complete dispersion of the material, the thin films of Mg‐PHI and Na‐PHI were deposited on an FTO substrate and dried at 50°C overnight. The incident light tests were measured using a Kessil LED Photoreaction Lighting PR160L source of 400 nm and intensity of 100 mW cm^−2^. Current density versus potential were conducted in a potential window from −0.55 to 1.3 V at a scan rate of 5 mV s^−1^ in 0.1 mol L^−1^ Na_2_SO_4_ supporting electrolyte. Saturated Calomel Electrode (SCE) and Platinum electrodes were used as reference and auxiliary, respectively.

#### 4.1.11 In Situ XANES

The X‐ray Absorption Near Edge Structure (XANES) spectra were acquired using an experimental ambient‐pressure XAS cell developed at APE‐HE beamline of the Elettra synchrotron radiation facility (Basoviozza Trieste, Italy). The set‐up allows analysis in the soft X‐ray range, using a Si_3_N_4_ membrane that separates the sample lodge at 1 bar from the rest of the beamline at UHV. The cell is interfaced with a temperature control system and a gas line whose flows are monitored by calibrated mass flow controllers. All reported spectra were obtained by continuous acquisition at 1 bar total pressure under real catalytic conditions. The X‐ray Absorption Spectroscopy (XAS) signal was recorded in Total Electron Yield (TEY) mode by measuring the drain current from the sample, making the technique inherently surface‐sensitive and enabling effective probing of relevant surface states.

### 4.2 Photocatalytic Hydrogen Evolution Reaction (HER)

The photocatalytic experiments were conducted in a sealed reactor, utilizing deionized water (DI) previously degassed and under Ar to avoid the presence of O_2_, an electron receptor that competes with proton reduction. A suspension composed of 27 mL of DI, 25 mg of solid catalyst and 3 mL of triethanolamine (TEOA), was subjected to irradiation using a 410 nm, 50 W LED lamp. The Pt cocatalyst was introduced in situ into the PHI's framework via photodeposition, utilizing a specific quantity of K_2_PtCl_4_ precursor. The first initial hour of irradiation, dedicated to Pt photodeposition, was excluded from hydrogen detection. The quantity of generated hydrogen was determined by monitoring the pressure within the reactor recorded by a digital pressure sensor P‐30 type manufactured by Wika.

The comparative analysis of photocatalyst performance was conducted based on the evolution rate and Apparent Quantum Yield (AQY), as determined using the equation provided above [52]:

$$AQY\;\left(\%\right)=\frac{N_{A}\times h\times c\times 2\times r_{H_{2}}}{S\times I\times \lambda }$$

where, *N_A_*, *h* and *c* are the Avogadro constant (6.022 × 10^23^ mol^−1^), Planck constant (6.626 ×  10^−34^ J s) and the speed of the light (3 ×  10^8^ m s^−1^), respectively. $r_{H_{2}}$ is the hydrogen evolution rate (mol h^−1^), *S* the irradiation area (12.25 cm^2^), *I* the irradiation light at the same interval (68.1 mW cm^−2^), and λ the wavelength of the monochromatic incident photons (m).

### 4.3 Mg‐PHI Model for the Excited States Simulation

In order to investigate the role of Mg^2+^ ions and the platinum nanoparticle on the photocatalysis process, a finite Na‐PHI model was built with a central pore surrounded by 6 pores and 30 Na^+^. Each Na^+^ was added up at the lowest free energy positions, using our lab‐made software called Themis [53], employing the Lennard–Jones parameters (taken from the CHARMM36 force field) [54] to describe the interactions. After that, 3 layers of this system were stacked using the same software to find the best rotation angle between the layers. Next, we used the Themis program to find and remove the two highest free energy Na^+^, resulting in a system with a net charge of −2 *e*. Then, another Themis run was perfomed to find the best position for a Mg^2+^ interacting with this system. This process was repeated until the number of added Mg^2+^ reached the experimental concentration of 5.5 wt.%, which corresponds to a 2:1 Mg^2+^/Na^+^ ratio, yielding a neutral 3‐layers Mg‐PHI system containing 36 Mg^2+^ and 18 Na^+^ ions. An icosahedral platinum nanoparticle consisting of 55 atoms was placed on the central pore with one of its vertices atoms placed at the pore's barycenter (Mg‐PHI‐NP model).

The Mg‐PHI/Pt model was placed at the center of a cubic box containing 9159 water molecules and 156 triethanolamine (TEOA) molecules, providing a realistic model for the excited states simulation in the presence of the sacrificial TEOA molecules in the experimental concentration (∼10% v/v). Using this system, a molecular dynamic simulation were carried out using the GROMACS suite [55], with the CHARMM36 force field^2^ describing the interactions. The simulations were performed using the following protocol: (1) energy minimization using the steepest descent algorithm; (2) 500 ps of NVT molecular dynamics simulation using the v‐rescale algorithm^5^ (T = 298.15 K, τ_T_ = 0.1 ps) to equilibrate the system; (3) 1 ns of a NPT step to equilibrate the pressure using the Berendsen barostat [56] (T = 298.15 K, τ_T_ = 0.1 ps, P = 1 bar, τ_P_ = 1.0 ps, κ = 4.6 × 10^−5^ bar^−1^); and (4) 200 ns of a NPT production run using the Parrinello–Rahman barostat [57] (T = 298.15 K, τ_T_ = 0.1 ps, P = 1 bar, τ_P_ = 5.0 ps, κ = 4.6 × 10^−5^ bar^−1^). In all molecular dynamic simulations, the center of mass motion was removed, and all bonds with hydrogen atoms were constrained using the fourth‐order LINCS algorithm [58].

The last structure obtained at the end of the molecular dynamic (MD) simulation was used to build the model for the excited states calculation. This model was built keeping only the cations that were inside the pores or in direct contact with the lateral edges of the PHI stack, as well as the water/TEOA molecules which were around each cation, inside the pores or around the nanoparticle. Since the Na^+^ and Mg^2+^ cations partitioned between the PHI pores and the surrounding solution, this resulting system had a net charge of −39 *e*, leading the quantum chemical calculation to a poor convergence and yielding UV–vis spectra in disagreement with the experimental result, so we neutralized the model system by adding 39 Na^+^ at the lowest free energy positions, with the help of our lab‐made software Themis.

The UV–vis spectrum with an energy threshold of 5 eV and Natural Transition Orbital (NTO) analysis were calculated with the stda software [59], which employs the simplified Tamm–Dancoff approximation, coupled with xtb4stda 1.0 software to generate the ground‐state wavefunction using the GFN1‐xTB semiempirical Hamiltonian [60]. Due to the small size of the model system compared to the experimental system, many non‐physical transitions are expected to occur. Therefore, all transitions with not well‐described intensities below 2.5 eV (above 496 nm) were removed from the theoretical spectrum. In addition, as many excited states can contribute to the same transition, the average NTO were plotted, using the following expression:

$$\psi =\sum_{n}{\left|c_{n}\right|}^{2}\psi_{n}$$

In which *c_n_* are the coefficients of each NTO wavefunction (ψ_*n*_), and ψ is the averaged NTO wavefunction. The sum of all the |*c_n_* |^2^ equals 1. Finally, Gabedit [61] software was used to generate the NTO cube files, and the VMD [62] software was used to plot the NTOs.

### 4.4 Synthetic Procedure

#### 4.4.1 Na‐PHI Synthesis

The synthesis of Sodium Poly(Heptazinic Imide)—Na(PHI) was carried out via ionothermal approach, where melamine, as nitrogen‐rich precursor, is pyrolyzed in the presence of a directing salt, herein NaCl [16]. Initially, a homogeneous solid mixture was obtained grinding melamine with NaCl, at a 1:10 (wt.%) ratio, using a ball mill for 10 min. The resulting fine powder was placed in an alumina crucible, loosely closed with a lid, and subjected to calcination at 600°C for 4 h, with a heating rate of 2.3° min^−1^, under N_2_ flow (0.2 L min^−1^). Subsequently, the resulting strong yellowish product was washed with deionized water (30 mL, 3 times) to remove the salt excess, isolated by centrifugation (9000 rpm, 15 min) and dried overnight at 60°C. The synthetic yield was approximately 60%–70%.

#### 4.4.2 Cationic Exchange

The incorporation of Mg^2+^ cations was achieved through direct cation exchange in aqueous medium, allowing the replacement of Na^+^ ions within the PHI framework by the desired metal. This method consists of suspending Na‐PHI in an aqueous solution containing a specific concentration of MgCl_2_. It enables controlled insertion of the new cation which depends solely on the solution concentration.

As reference, for the preparation of Mg‐PHI containing 5.5% of Mg^2+^ incorporated, 100 mg of Na‐PHI was initially suspended in 2 mL of an aqueous MgCl_2_·6H_2_O solution (5 m) (see Table 1 for other concentrations). The mixture was then sonicated for 15 min, washed with deionized water (2 mL, 3 times), afterward, the powder was isolated through centrifugation (13 500 RPM, 5 min) and dried overnight at 60°C.

## Funding

This research was financially supported by the Brazilian funding agencies CAPES, CNPq (423196/2018, 403064/2021, 307043/2022‐3, 302607/2026‐9 and 405752/2022), FINEP (01.23.0645.00 and 01.23.0662.00) and FAPESP (2021/140067, 2023/04252‐6, 2020/14741, 2021/13271, 2022/16273, 2021/12394, 2022/14652, 2024/02739‐8 and 2021/11162). The authors acknowledge financial support from Max Planck Society and the German Research Foundation (DFG) via the Cluster of Excellence e‐conversion (2089/1‐390776260). This project received funding from the European Union's Horizon Europe research and innovation program under grant agreement no. 101046836 (project acronym “CATART”). The publication reflects only the author's views and the European Union is not liable for any use that may be made of the information content therein. SS acknowledges financial support by the CATART project via a Ph.D. grant. We acknowledge support from UniPV and MUR through the program ‘Departments of Excellence’ (2023‐2027).

## Conflicts of Interest

The authors declare no conflicts of interest.

## Supporting information




**Supporting File**: smll75403‐sup‐0001‐SuppMat.pdf.

## Data Availability

The data that support the findings of this study are available from the corresponding author upon reasonable request.
