# Supplementary material for: Mg2+ Ions as Interfacial Charge Bridges for Enhanced Photocatalytic Hydrogen Evolution in Poly(Heptazine Imide)
Source: Small. 2026 Aug 27;22(61):e75403. doi: 10.1002/smll.75403 (PMC13725419; doi:10.1002/smll.75403)
Supplement: Supplementary file 1 [file SMLL-22-e75403-s001.pdf]

Supporting Information

Mg^{2+} Ions as Interfacial Charge Bridges for Enhanced Photocatalytic Hydrogen Evolution in Poly(Heptazine Imide)

*Gabriel A. A. Diab, Vitor G. S. Pastana, Sara Stolfi, Rayse M. Ferreira, Izadora F. Reis, Marcos A. R. da Silva, Caio V. C. Ribeiro, Kathrin Küster, Piero Torelli, Paolo Ghigna, Viola Duppel, Maurizio Fagnoni, Sebastian Bette, Bruna R. Serino, Andre F. de Moura, Davide Ravelli, Bettina V. Lotsch, Ivo F. Teixeira**

Contents

1. Supplementary Data 3

Elemental Composition.. 3

Thermogravimetric Analysis (TG/DTA) 4

Simulated PXRD data 5

X-Ray Photoelectron Spectroscopy 6

In situ photoluminescence experiments 8

Electro- and Photoelectrochemical experiments.. 12

Transmission Electron Microscopy 15

Post-Reaction Characterization.. 24

Molecular Dynamics Simulations.. 25

References.. 28

1. Supplementary Data

Elemental Composition

Table S1. Elemental composition of each fabricated photocatalyst measured through elemental analysis and ICP-OES.

Label	N (Wt. %)	C (Wt. %)	H (Wt. %)	Na^+ (Wt. %)	Mg^{2+} (Wt. %)	C/N ratio
Na-PHI	43.22	26.67	2.58	6.62	-	0.72
Mg-PHI 3%	43.98	27.07	2.85	5.87	2.66	0.72
Mg-PHI 4%	43.90	27.05	2.82	5.78	4.17	0.72
Mg-PHI 4.5%	44.38	27.24	3.06	3.77	4.58	0.72
Mg-PHI 5.5%	44.07	27.08	3.18	2.91	5.42	0.72

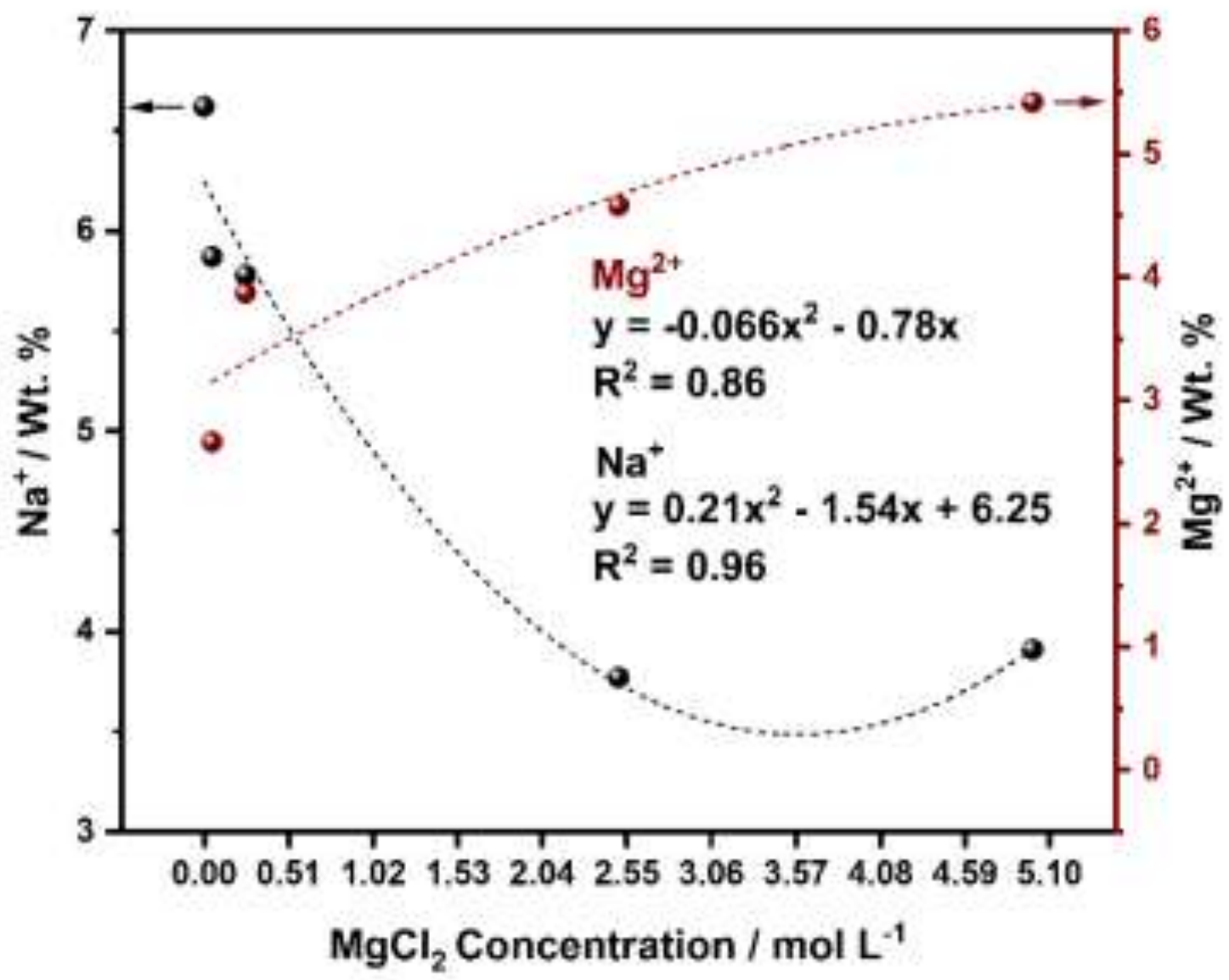

Figure S1. Relationship between the loading curves of each cation content and the $MgCl_2$ solution concentration. The contents of both Na^+ and Mg^{2+} were fitted using a quadratic regression model using the ICP-OES data.

Thermogravimetric Analysis (TG/DTA)

Thermal decomposition experiments reveal that both Na- and Mg-PHI exhibit thermal stability up to approximately 650 °C. Beyond this temperature, a sharp mass loss is observed, corresponding to the irreversible collapse of the PHI carbon nitride framework. These profiles indicate that the incorporation of Mg^{2+} does not compromise the thermal integrity of the polymer. Regarding the hydration state, both materials display a weight loss of roughly 17 wt%, attributed to the release of hydration water molecules coordinated to the intrapore cations.

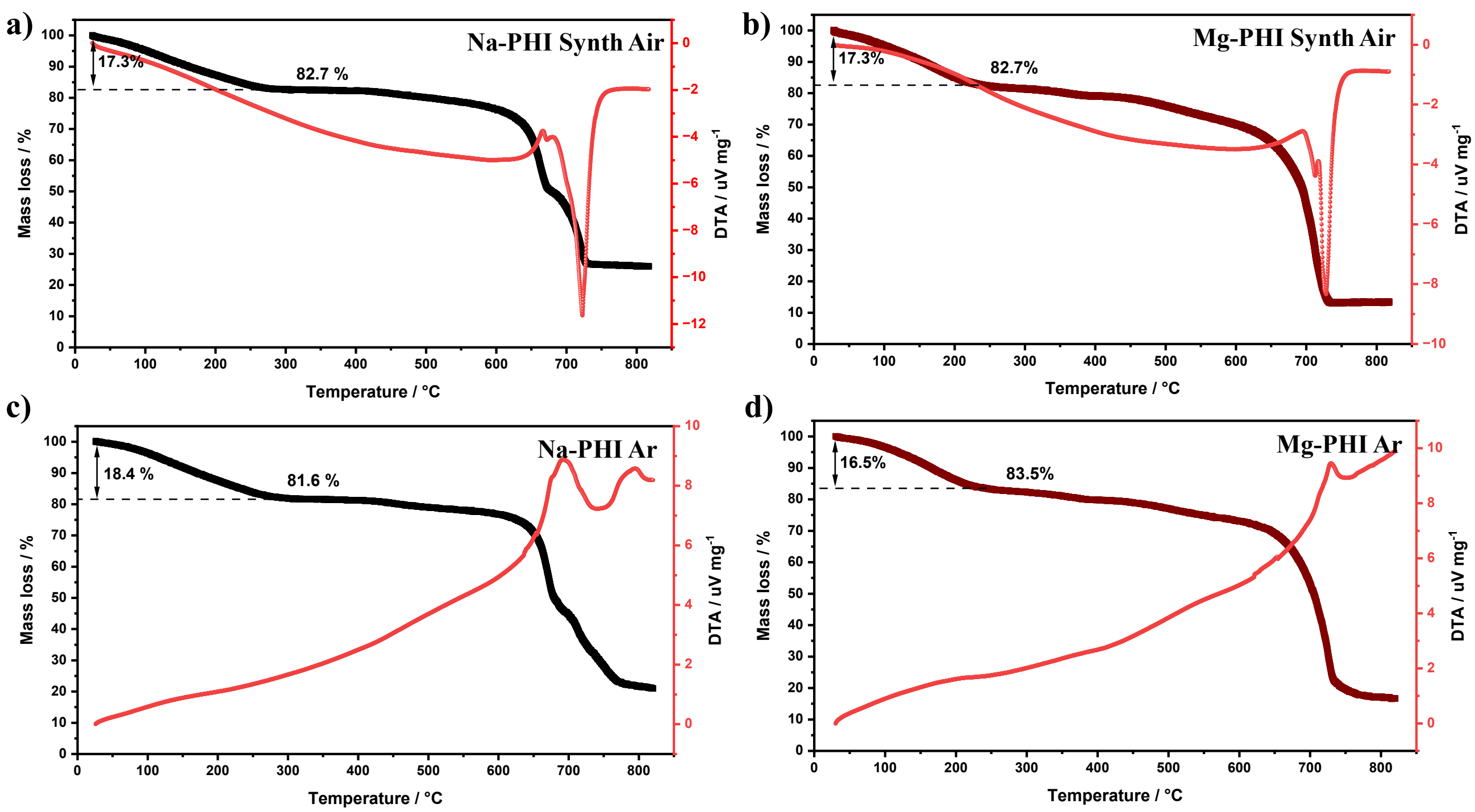

Figure S2. Thermogravimetric analysis (TG and DTG) of Na- and Mg-PHI 5.5% under different atmosphere: **a-b)** synthetic air and **c-d)** Ar.

Simulated PXRD data

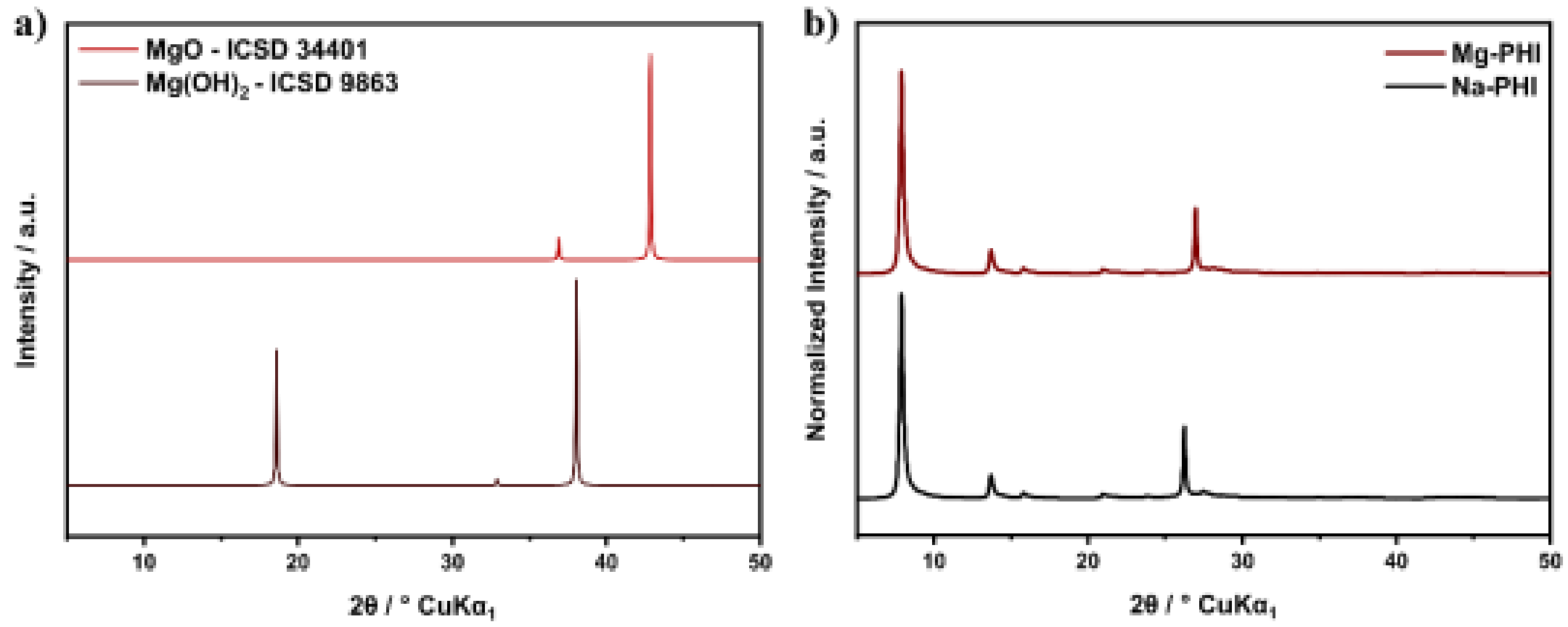

Figure S3. Simulated XRD patterns for **a)** the oxygenated phases of Mg - MgO and $Mg(OH)_2$ and **b)** a simplified and disordered structure for Na-PHI and its counterpart containing Mg^{2+} ions.

X-Ray Photoelectron Spectroscopy

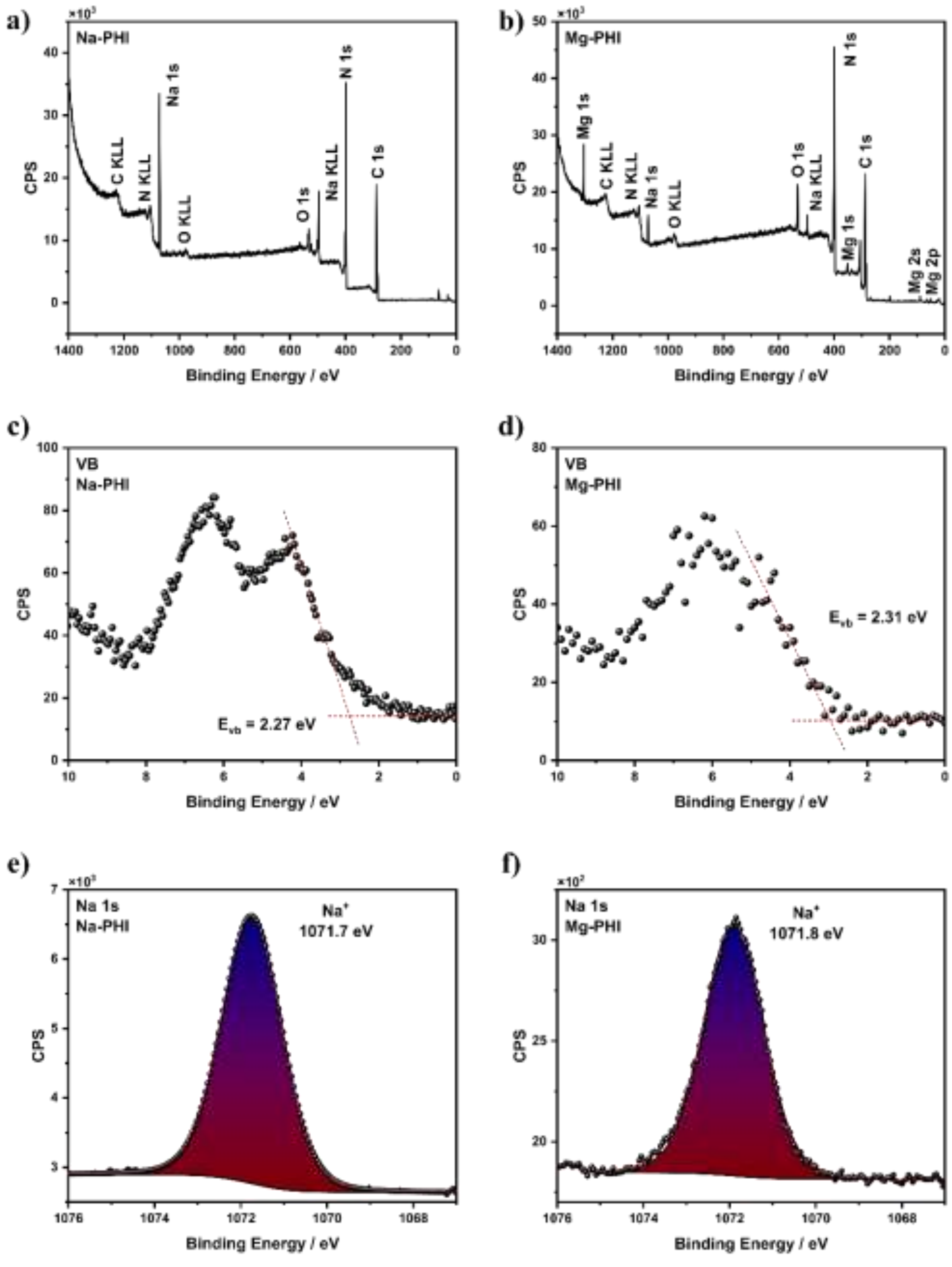

Figure S4. Additional XPS data of Na-PHI and Mg-PHI 5.5. **a-b)** Survey spectra of Na-PHI and Mg-PHI, respectively; **c-d)** the corresponding spectra of core levels to the valence band maximum of each material; **e-f)** High resolution XPS in the Na 1s region of both materials.

Table S2. Quantitative XPS for Na-PHI extracted from the survey given in atomic percentage (at%).

O 1s	C 1s	N 1s	Na 1s
5.2%	43.8%	45.0%	6.0%

Table S3. Quantitative XPS for Mg-PHI 5.5% extracted from the survey given in atomic percentage (at%).

O 1s	C 1s	N 1s	Cl 2p	Mg 1s	Na 1s
7.1%	46.4%	43.7%	1.1%	1.1%	0.7%

In situ photoluminescence experiments

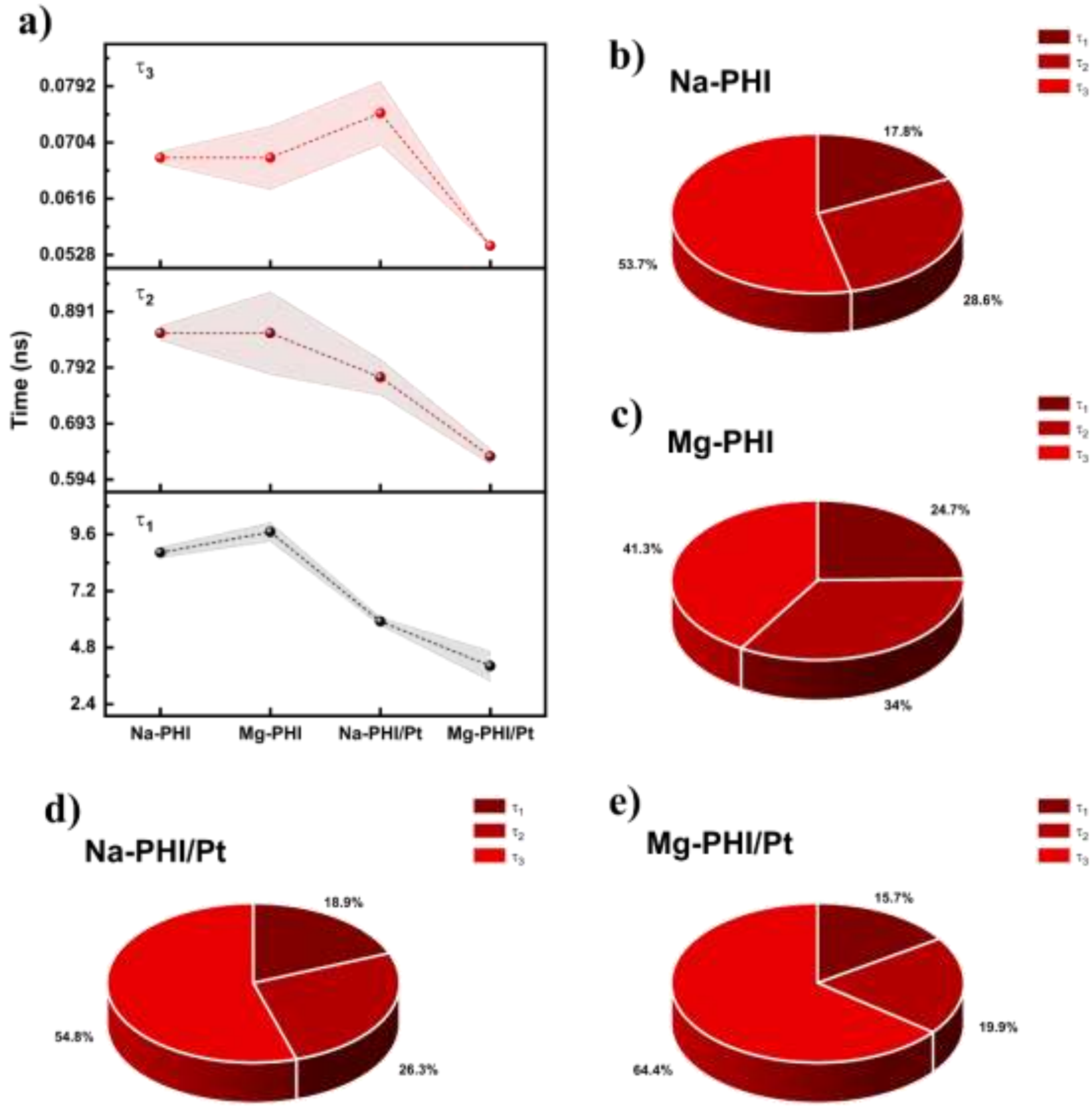

Figure S5. a) progression of the lifetime components according to each sample; **b–e)** relative contribution of lifetimes (τ_1, τ_2, τ_3) for each sample.

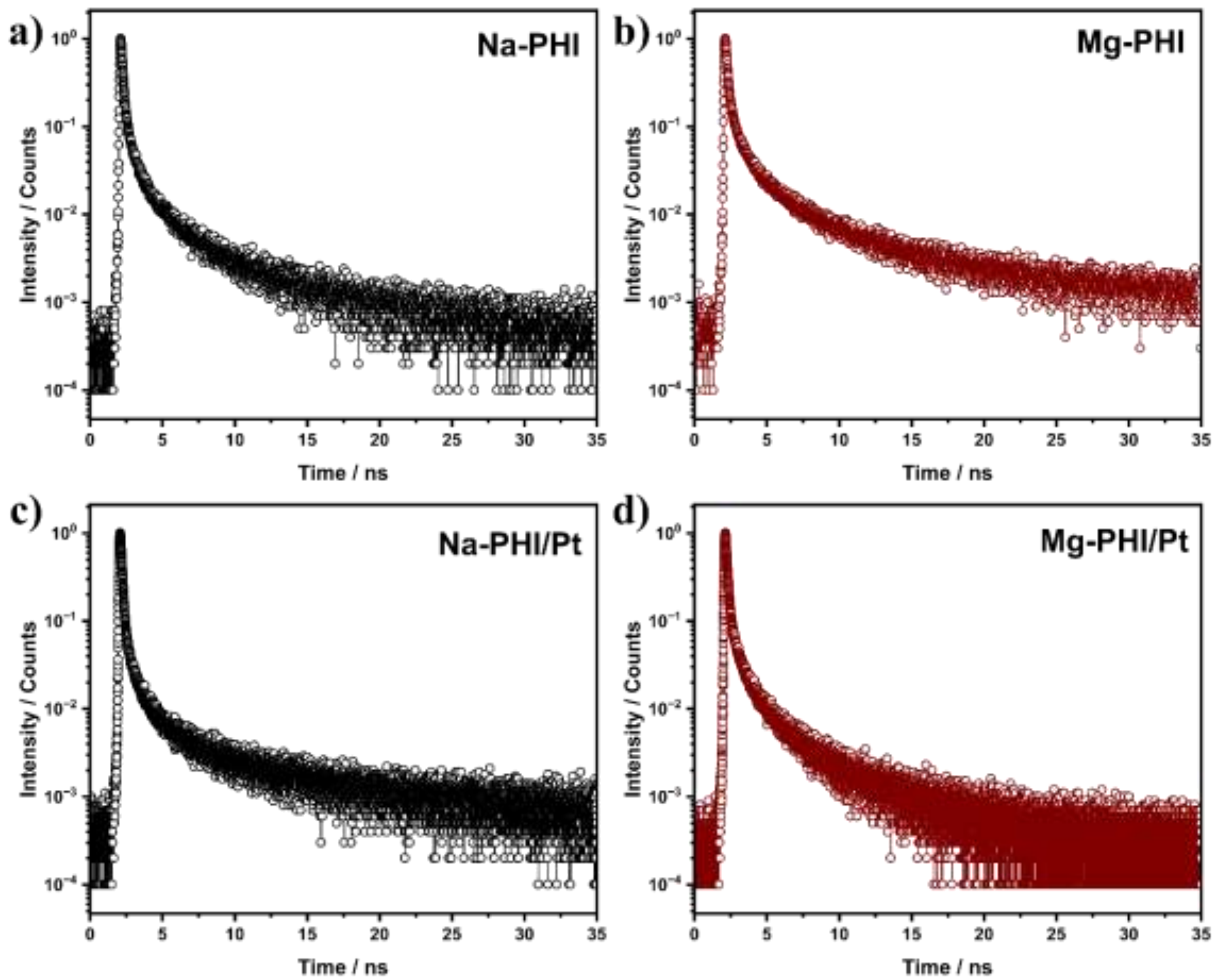

Figure S6. Individual time-resolved photoluminescence plot for **a)** Na-PHI, **b)** Mg-PHI and **c-d)** their Pt-containing counterparts in the logarithmic scale.

Table S4. TR-PL parameters obtained from the fitting of the decay curves for Na-PHI, where A represents the amplitude (kCnts/Chnl), τ the lifetime of each component (ns), and I the time-dependent intensity function (kCnts).

Parameter	Value	Absolute error / Δ	Relative Error / δ
A_1	0.056	0.002	3.2
τ_1	8.83	0.22	2.4
I_1	122.3	1.4	1.1
A_2	0.730	0.012	1.6
τ_2	0.853	0.013	1.4
I_2	155.6	1.1	0.7
A_3	29.41	0.26	0.9
τ_3	0.068	0.001	0.9
I_3	502.8	3.0	0.6

**B_{kgrDec}**	0.0048	0.0004	6.7
**B_{kgrIRF}**	1.13	0.53	46
**$Shift_{IRF}$**	-44.68	0.36	0.8
τ_{Av}	1.597	0.021	1.3
χ^2	1.689	-	-

Table S5. TR-PL parameters obtained from the fitting of the decay curves for Mg-PHI, where A represents the amplitude (kCnts/Chnl), τ the lifetime of each component (ns), and I the time-dependent intensity function (kCnts).

Parameter	Value	Absolute error / Δ	Relative Error / δ
**A_1**	0.133	0.009	6.7
τ_1	9.70	0.40	4.1
**I_1**	122.3	4.4	2.7
**A_2**	0.730	0.096	6.7
τ_2	0.853	0.073	5.8
**I_2**	155.6	1.7	0.7
**A_3**	29.41	1.2	5.3
τ_3	0.068	0.005	5.4
**I_3**	269.8	6.0	2.2
**B_{kgrDec}**	0.0067	0.0001	1.1
**B_{kgrIRF}**	2.20	0.24	11
**$Shift_{IRF}$**	-20.7	2.1	10
τ_{Av}	2.860	0.054	1.9
χ^2	1.848	-	-

Table S6. TR-PL parameters obtained from the fitting of the decay curves for Na-PHI/Pt, where A represents the amplitude (kCnts/Chnl), τ the lifetime of each component (ns), and I the time-dependent intensity function (kCnts).

Parameter	Value	Absolute error / Δ	Relative Error / δ
**A_1**	0.113	0.006	5.6
τ_1	5.91	0.17	2.8
**I_1**	83.0	2.2	2.6
**A_2**	1.378	0.058	4.2
τ_2	0.775	0.032	4.1
**I_2**	133.4	1.2	0.9
**A_3**	26.8	1.6	5.6
τ_3	0.075	0.005	6.2
**I_3**	250.5	3.3	1.3
**B_{kgrDec}**	0.0038	0.0001	1.7
**B_{kgrIRF}**	1.11	0.39	35

**Shift$_{IRF}$**	-36.0	2.2	5.9
$\boldsymbol{\tau_{Av}}$	1.310	0.024	1.8
$\boldsymbol{\chi^2}$	1.752	-	-

Table S7. TR-PL parameters obtained from the fitting of the decay curves for Mg-PHI/Pt, where A represents the amplitude (kCnts/Chnl), τ the lifetime of each component (ns), and I the time-dependent intensity function (kCnts).

Parameter	Value	Absolute error / Δ	Relative Error / δ
**A$_1$**	0.155	0.005	3.3
$\boldsymbol{\tau_1}$	4.014	0.063	1.5
**I$_1$**	311.9	4.6	1.5
**A$_2$**	1.366	0.032	2.3
$\boldsymbol{\tau_2}$	0.635	0.014	2.2
**I$_2$**	433.1	3.8	0.9
**A$_3$**	33.30	0.29	0.9
$\boldsymbol{\tau_3}$	0.0542	0.001	1.9
**I$_3$**	902.1	5.4	0.6
**B$_{kgrDec}$**	0.0032	0.0001	1.4
**B$_{kgrIRF}$**	0.66	0.13	19
**Shift$_{IRF}$**	-42.66	0.61	1.4
$\boldsymbol{\tau_{Av}}$	0.957	0.007	0.7
$\boldsymbol{\chi^2}$	1.646	-	-

Electro- and Photoelectrochemical experiments

We extended the investigation to observe the proposed interface by the electro- and photoelectrochemical perspective. Therefore, a series of electrochemical characterizations were performed comparing the materials before and after the reaction, *i.e.* with and without Pt-co-catalyst. All measurements were performed in neutral aqueous solution containing 0.1 mol L^{-1} Na_2SO_4 as electrolyte. These experiments aimed to evaluate the onset potential, photocurrent density, stability under illumination, and interfacial charge transfer characteristics of the photoelectrodes deposited on FTO substrates.

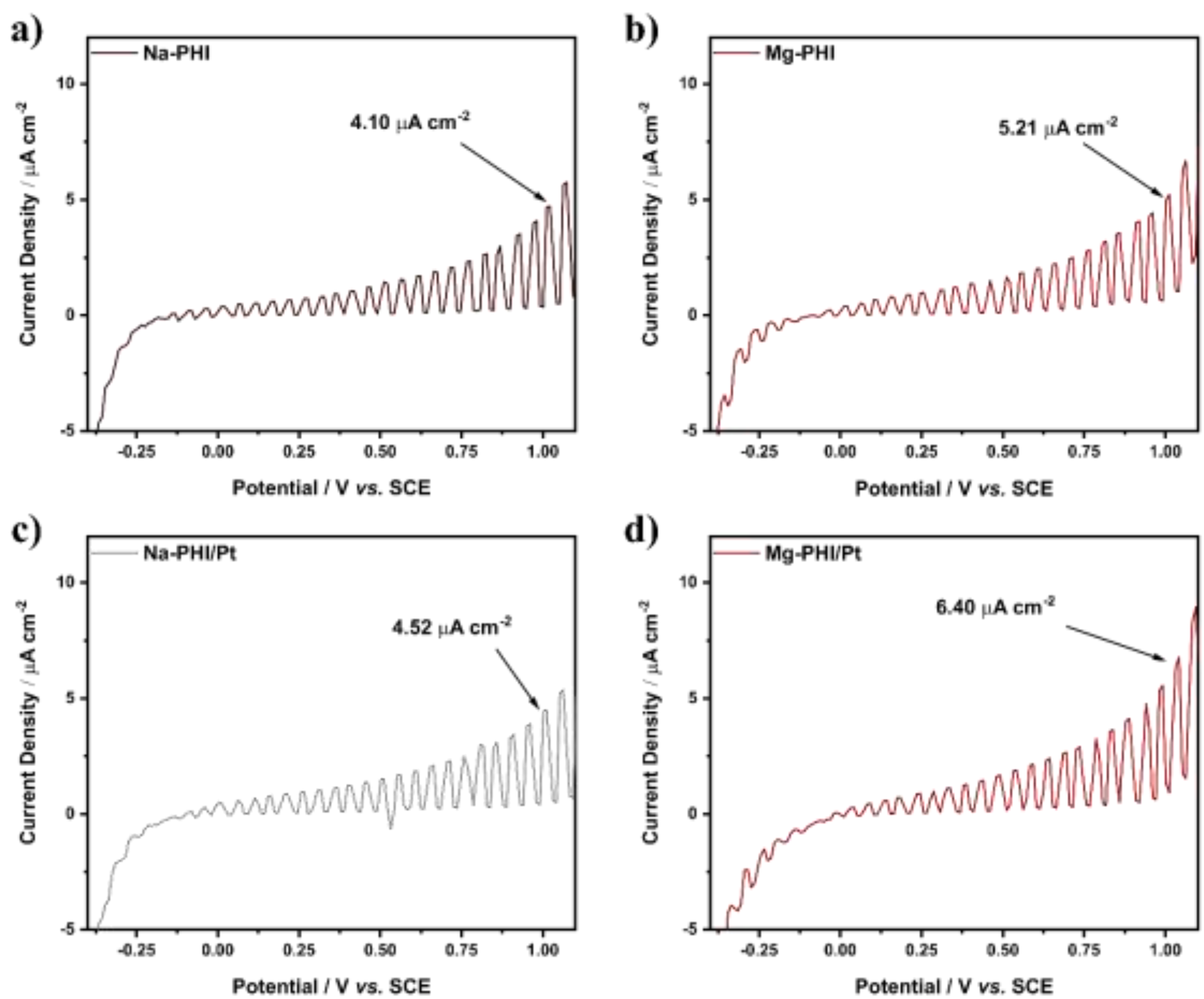

Figure S7. LSV curves of photoelectrodes at a scan rate of 5 mV s^{-1}, 0.1 mol L^{-1} Na_2SO_4 upon on/off illumination of FTO/M-PHI.

Initially, linear sweep voltammetry (LSV) measurements under chopped visible light (400 nm, 5 s ON / 5 s OFF) revealed the photoresponse of each material (**Fig. S7 and S8a**). All photoelectrodes exhibited a negative onset potential for photocurrent generation, below 0 V *vs* SCE, which is a characteristic behavior of inorganic semiconductors.[17] A rapid increase in photocurrent was observed for all materials, sustained over a wide potential window, evidence of relevant photoelectrocatalytic activity. Importantly, Mg-PHI containing Pt (Mg-PHI/Pt) exhibited the highest photocurrent density (6.40 μA cm^{-2}), outperforming its counterpart without Pt (5.21 μA cm^{-2}) as well as the reference materials Na-PHI (4.10 μA cm^{-2}) and Na-PHI/Pt (4.52 μA cm^{-2}), while showing no signs of photocorrosion.

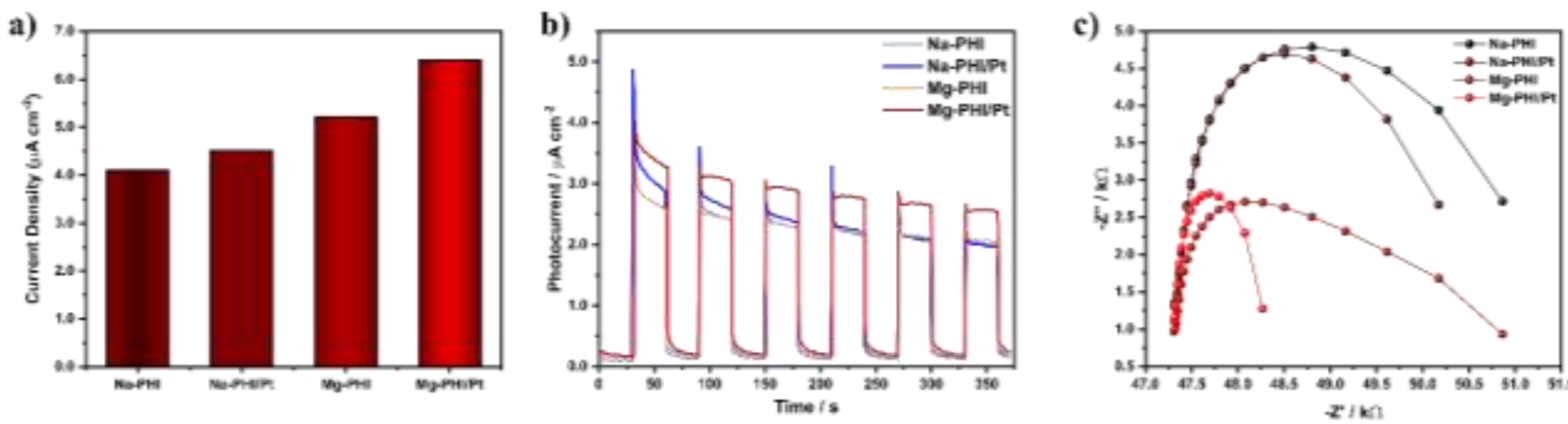

Figure S8. Photoelectrochemical response of Na- and Mg-PHI 5.5% and their counterparts containing Pt electrodes deposited on FTO. **a)** Current density determined by Linear Sweep Voltammetry (LSV) extracting the values at 1 V *vs* SCE recorded at a scan rate of 5 mV s^{-1} in 0.1 mol L^{-1} Na_2SO_4 under chopped ON/OFF illumination; **b)** Transients chronoamperometry for different photoelectrodes under chopped illumination (t_{ON} = t_{OFF} = 30 s) in 0.1 mol L^{-1} Na_2SO_4 electrolyte; **c)** Comparative Nyquist plots of the same electrodes.

Chronoamperometry (CA) measurements under intermittent light (t_{ON} = t_{OFF} = 30 s) at 1.0 V *vs* Saturated Calomel Electrode (SCE) (**Fig. 8b**). The Mg-PHI/Pt electrode achieved an average photocurrent of 2.97 ± 0.42 μA cm^{-2}, while Na-PHI, Na-PHI/Pt, and Mg-PHI photoelectrodes exhibited slightly lower values of 2.30 ± 0.26, 2.43 ± 0.40, and 2.32 ± 0.23 μA cm^{-2}, respectively. These results are consistent with the enhanced performance previously observed in the LSV experiments. This improvement indicates superior charge separation and transport capabilities in the Pt-containing materials, with Mg-PHI/Pt standing out. The presence of Pt nanoparticles at the sheet edges appears to modulate the final charge carrier dynamics, either by generating a local

electric field that facilitates charge separation and transport, or by acting as trap states that stabilize photogenerated charges.[18]

In addition to higher photocurrent values, some electrodes exhibited improved stability during prolonged illumination (**Fig. S8b**). Again, the materials containing Pt demonstrated higher stability in the photocurrent profiles over time, with photocurrent decay of less than 68% and 64% for Na-PHI/Pt and Mg-PHI/Pt, respectively. In contrast, Na-PHI and Mg-PHI showed larger losses (74% and 76%), suggesting greater recombination of photogenerated charge carriers on those surfaces. The pronounced spikes observed upon illumination indicate surface recombination phenomena, confirming that part of the photogenerated holes is not efficiently consumed in the redox process.[19]

One way to explain the photocurrent results obtained from the different samples is through charge transfer resistivity analysis. Such results can be correlated with the electrochemical impedance spectroscopy (EIS) measurements in the dark environment that were performed to determine the internal resistance values. The measurements were conducted at open circuit potential (OCP), with an amplitude of 10 mV, in a frequency range of 0.1-1e^5 Hz. The spectra were fitted using the Randles equivalent electrical circuit , which comprises the electrolyte resistance (R_S approximately 43 Ω) in series with a parallel combination of interfacial charge transfer resistance (R_{CT}) and constant phase element (CPE). The Nyquist diagrams of the photoelectrodes exhibit very different profiles (**Fig. S8c**). The profiles reveal distinct semicircle shapes for each sample, indicating variations in interfacial charge transfer resistances. The R_{CT} values for the Na-PHI, Na-PHI/Pt, Mg-PHI, and Mg-PHI/Pt photoelectrodes were 18.5, 8.83, 4.79, and 3.12 kΩ, respectively. These values reflect the inherently high resistivity of carbon nitride-based materials, as previously reported in the literature.[20, 21] The nearly complete semicircle observed in all spectra further corroborates this characteristic. Notably, a general decrease in R_{CT} was observed across all samples containing Pt. This reduction in resistance is attributed to the formation or activation of catalytic sites, particularly those associated with transition metals such as Pt, during the reaction. Among the tested materials, the Mg-based photoelectrodes exhibited the most significant decrease in semicircle diameter, particularly Mg-PHI/Pt, indicating a more efficient interfacial charge transfer. These results are in concordance with the photocurrent densities observed in the LSV measurements, further validating the superior photoelectrocatalytic performance of the Mg-modified samples.

Transmission Electron Microscopy

The morphological and compositional features of the materials were analyzed using Transmission Electron Microscopy (TEM) imaging and Energy-Dispersive X-ray spectroscopy (EDX). The data for the Mg-containing material, its precursor Na-PHI and their Pt-loaded counterparts are displayed in **Fig. S9-S11**. The images revealed the characteristic long sheet-like morphology with a primary structure of large and agglomerated flakes with extension of approximately 1500 nm. Repetition patterns or alignment fringes are clearly observed in the images, extending across the sheets in a wave-like distribution originating from a well-defined central point, a feature not commonly observed in other semi-crystalline carbon nitride materials reported in the literature.[22] The same pattern is also evident in the Mg^{2+} containing samples. Notably, in both cases, as highlighted by the Fast Fourier Transforms (FFT) shown in the inset, the produced material is highly ordered, where semicircles patterns associated with the 100 and 110 reflections are clearly seen. The incorporation of magnesium does not significantly alter the morphological features of the material. Similarly, no evident morphological alterations were observed following the photodeposition of the Pt co-catalyst. Notably, the Pt nanoparticles were deposited at well-defined sites within the PHI framework rather than being homogeneously distributed across the sheets. This preferential deposition likely occurs at cyanamide defect sites, as previously reported by Lotsch *et al.* for modified PCN materials.[23]

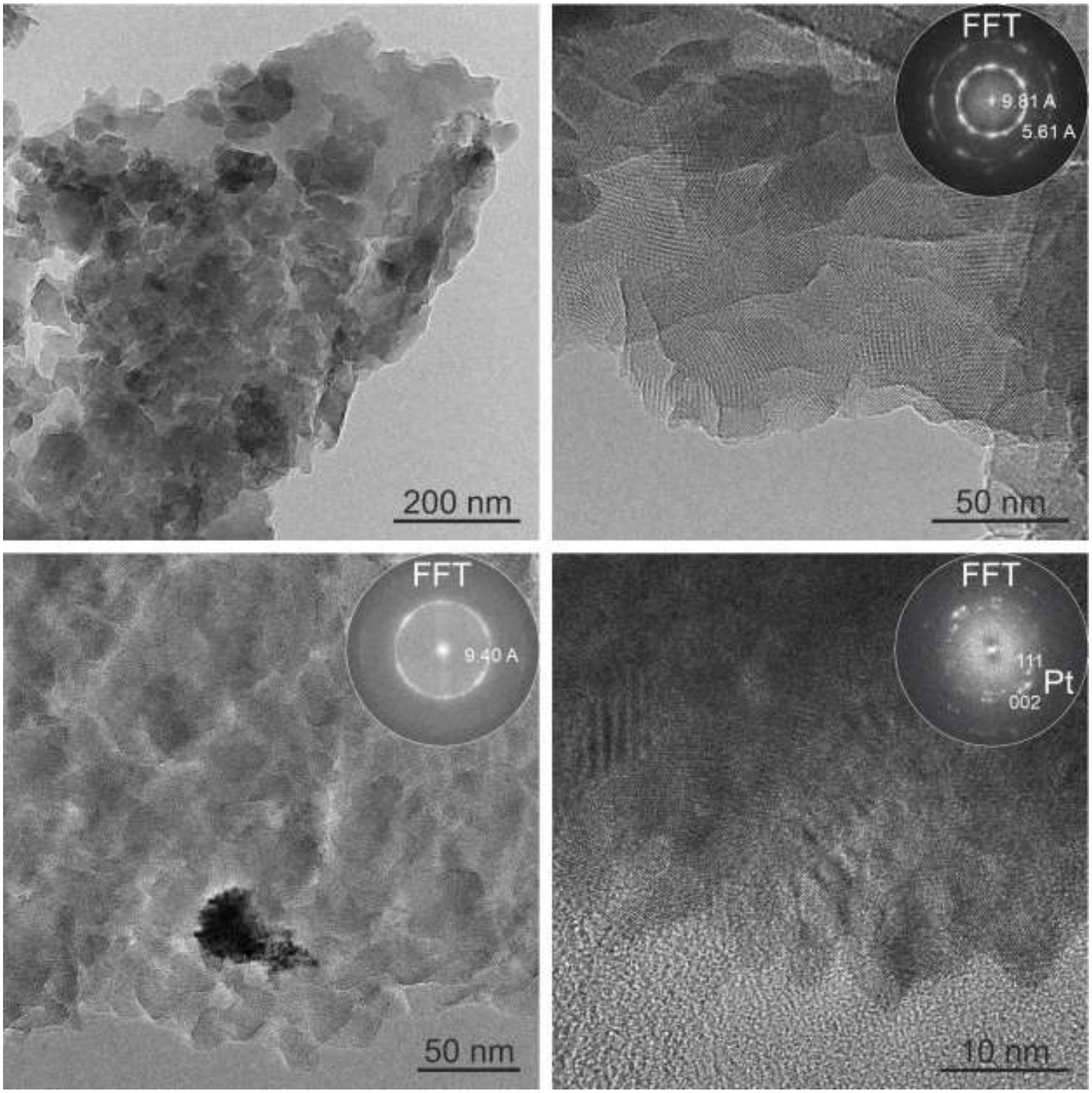

Figure S9. TEM images of Na-PHI photocatalyst before (top row) and the same material after Pt photodeposition (bottom row). The corresponding Fast Fourier Transform (FFT) are shown in the insets.

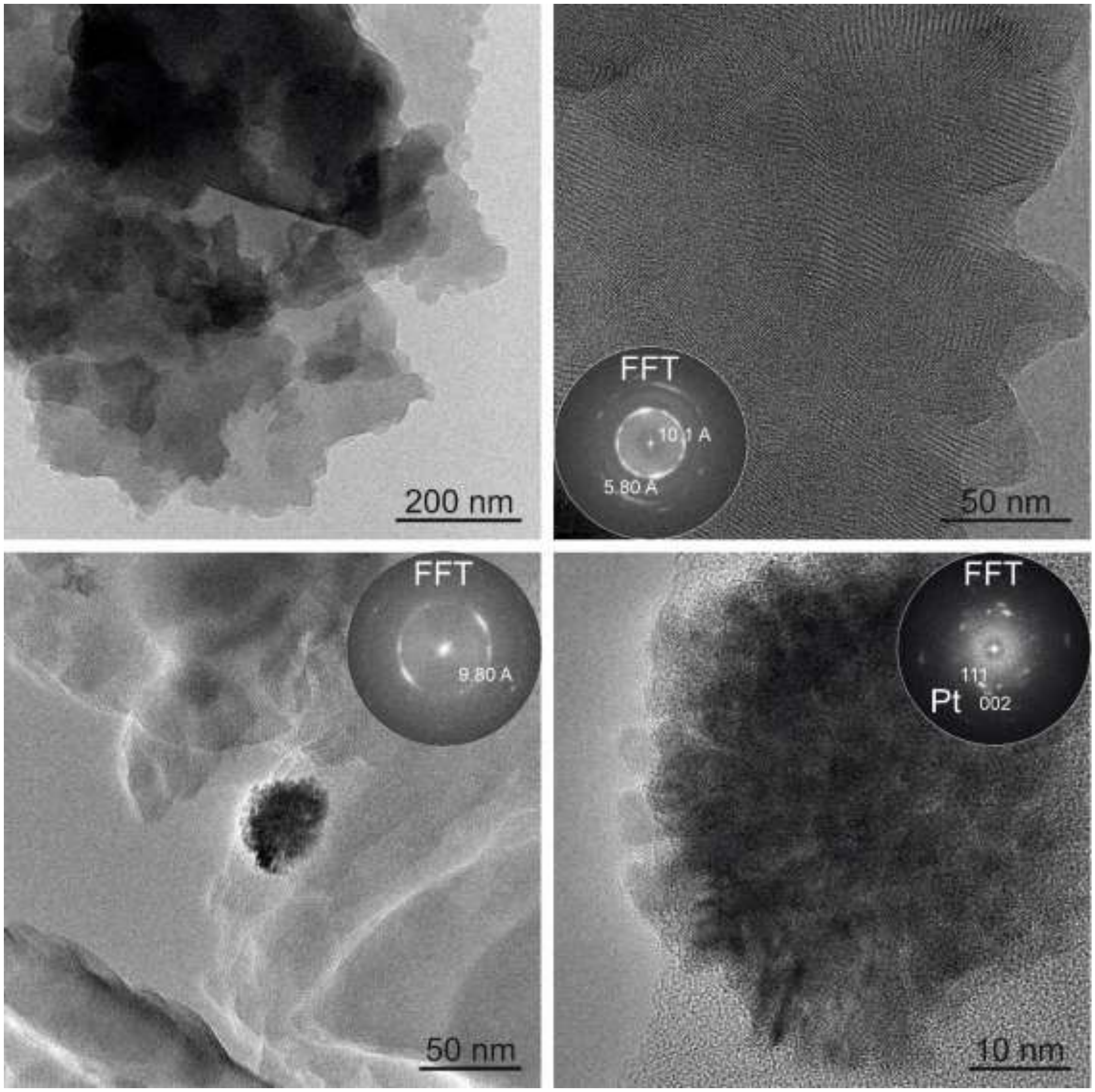

Figure S10. TEM images of the fresh Mg-PHI 5.5% photocatalyst (top row) and the same material after Pt photodeposition (bottom row). The corresponding Fast Fourier Transforms (FFT) are shown in the insets.

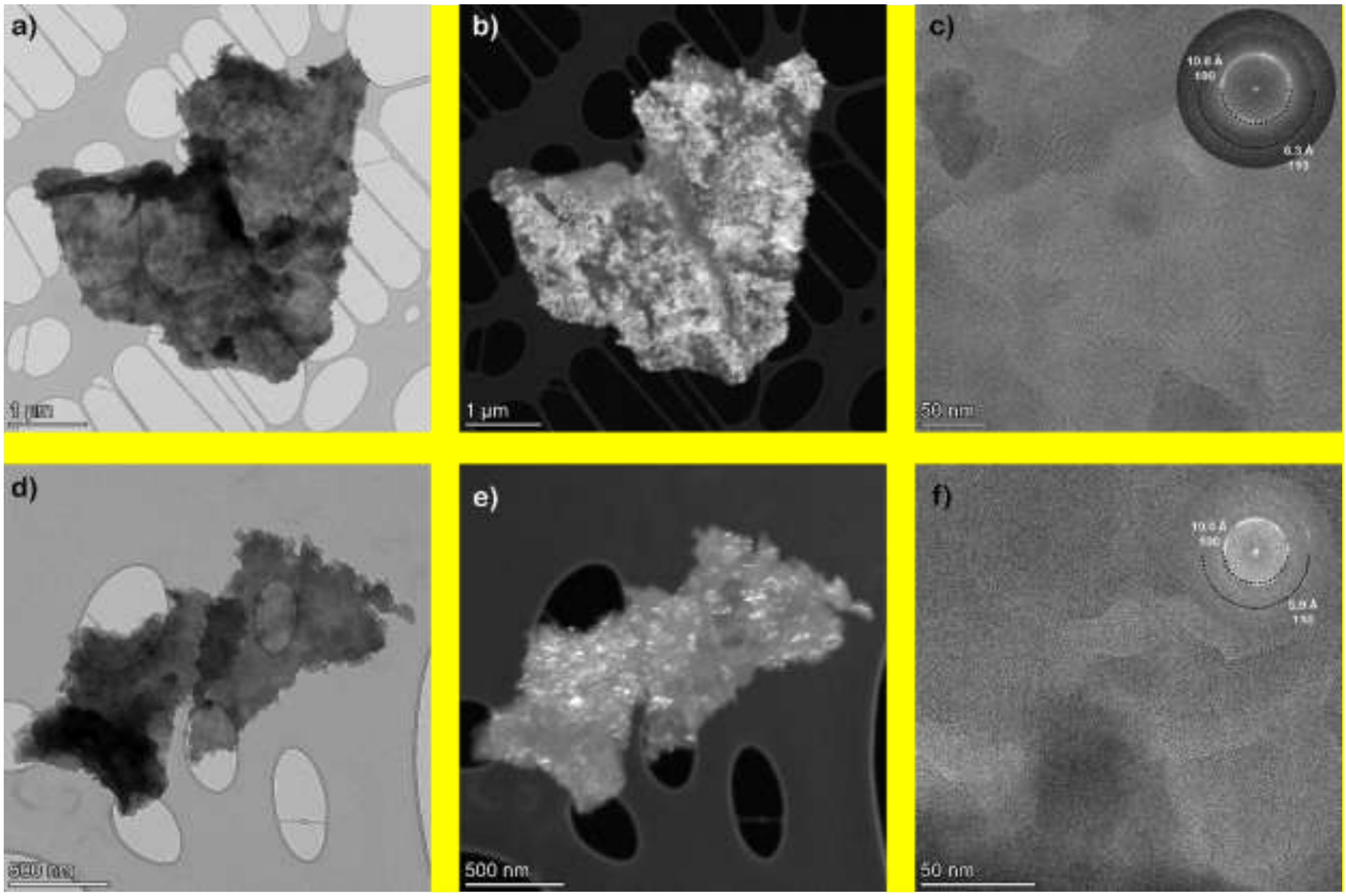

Figure S11. TEM images comparison between fresh Na-PHI (**a–c**) and Mg-PHI 5.5% (**d–f**) photocatalysts. Images **a)** and **d)** were acquired using the transmitted beam, while **b)** and **e)** dark field TEM images at high diffraction angle. The bright-contrast regions correspond to crystalline domains responsible the {100}. High-resolution TEM (HRTEM) images are shown in **c)** and **f)**.

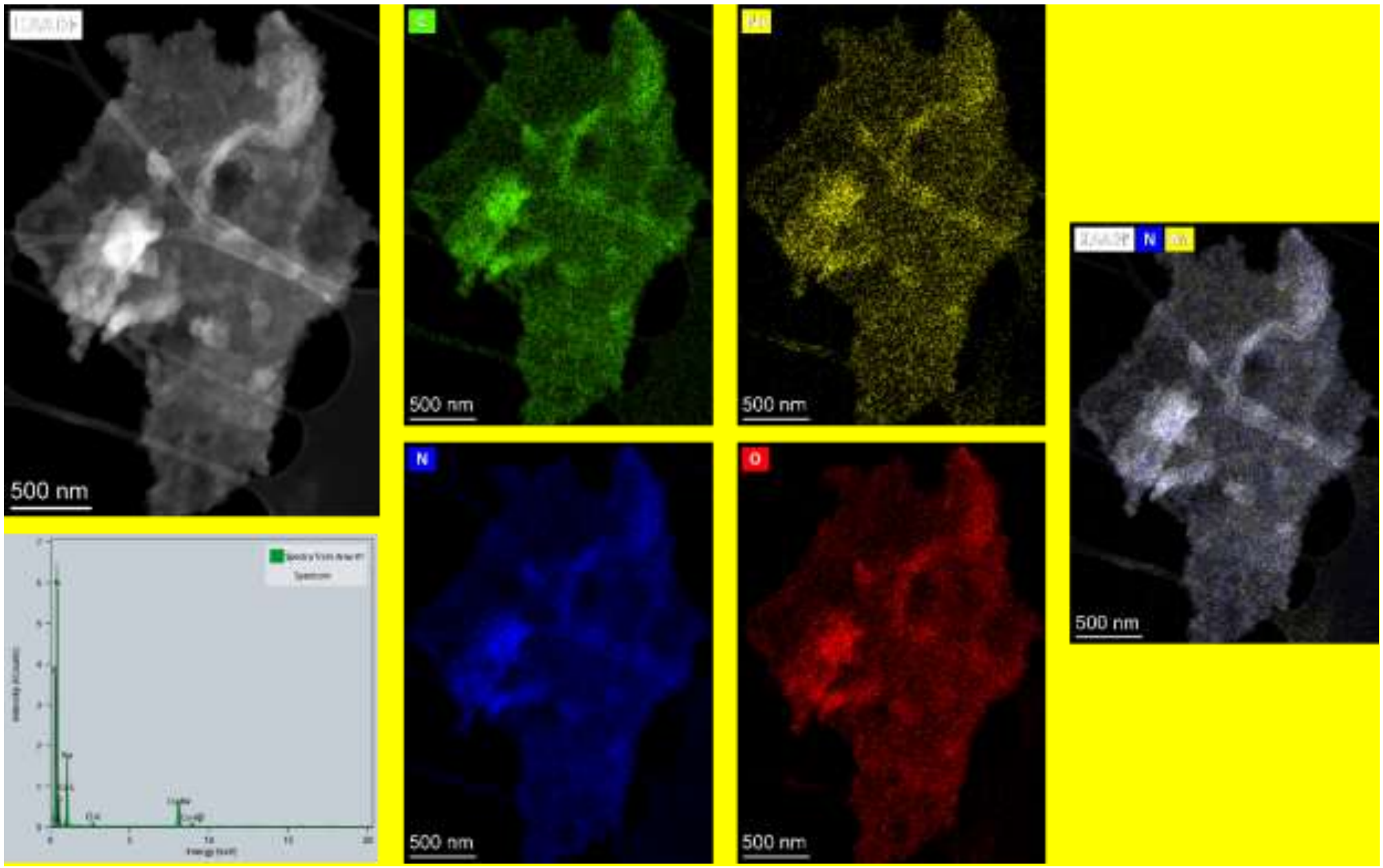

Figure S12. EDX mapping images of the fresh Na-PHI photocatalyst.

Table S8. Quantitative compositional analysis of Na-PHI extracted from TEM.

Element	Atomic Fraction / %	Mass Fraction / %
**C**	41.1 (3.8)	31.8 (2.8)
**N**	45.1 (5.0)	40.7 (4.9)
**O**	3.3 (0.7)	3.3 (0.7)
**Na**	7.1 (1.5)	10.5 (2.1)

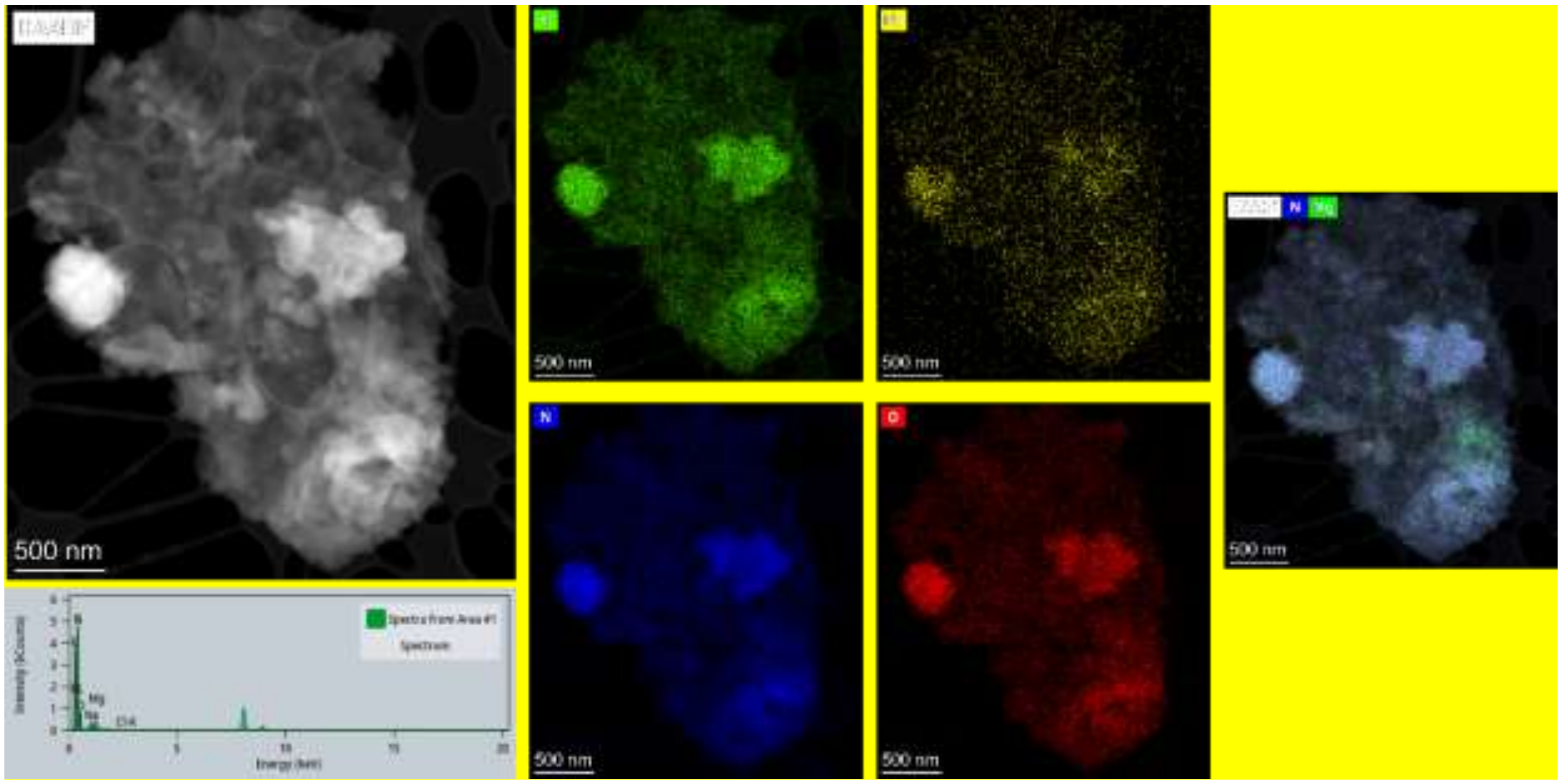

Figure S13. EDX mapping images of the fresh Mg-PHI 5.5% photocatalyst.

Table S9. Quantitative compositional analysis of Mg-PHI/Pt extracted from TEM.

Element	Atomic Fraction / %	Mass Fraction / %
**C**	48.1 (4.2)	42.8 (3.8)
**N**	42.9 (4.9)	44.6 (4.9)
**O**	5.5 (0.9)	6.5 (1.4)
**Na**	1.7 (0.3)	2.8 (0.6)
**Mg**	1.8 (0.3)	3.2 (0.7)

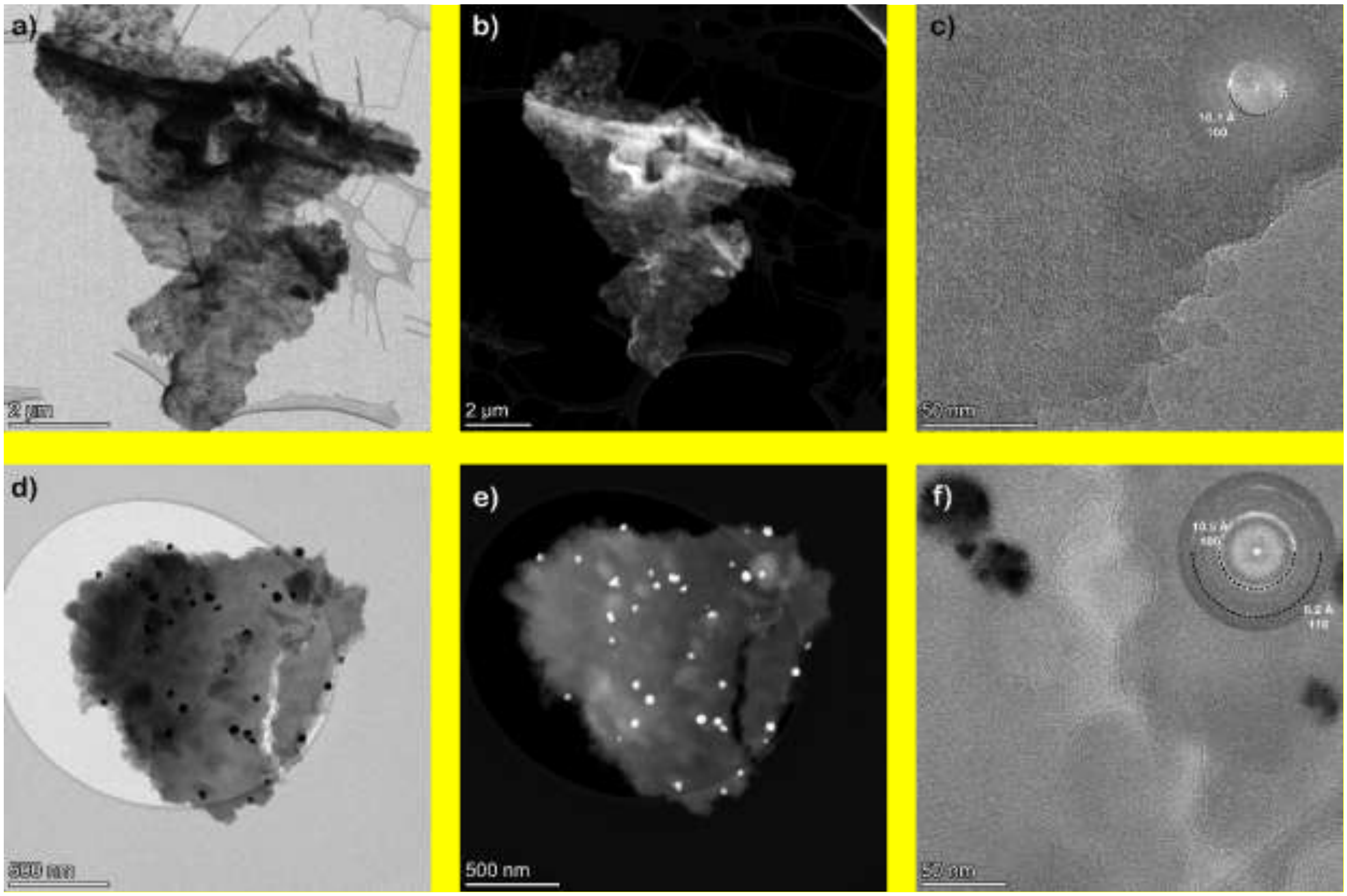

Figure S14. Bright Field and Dark Field STEM images comparison between fresh Na-PHI/Pt (**a–c**) and Mg-PHI/Pt (**d–f**) photocatalysts. High-resolution TEM (HRTEM) images are shown in **c)** and **f)**.

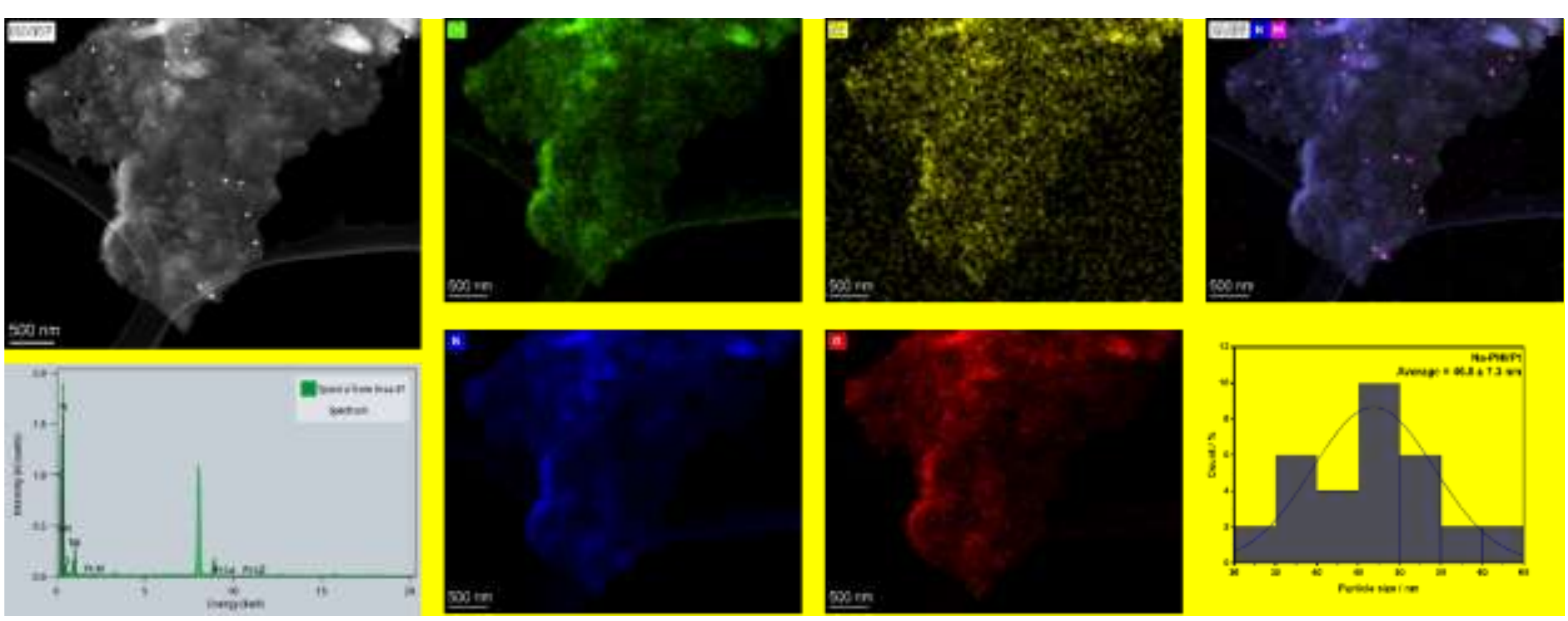

Figure S15. EDX mapping images of the Na-PHI/Pt photocatalyst. The last panel represents the size distribution of the Pt nanoparticles.

Table S10. Quantitative compositional analysis of Na-PHI/Pt extracted from TEM.

Element	Atomic Fraction / %	Mass Fraction / %
**C**	45.7 (4.5)	40.2 (4.1)
**N**	47.6 (5.1)	48.8 (5.1)
**O**	1.9 (0.4)	2.3 (0.5)
**Na**	4.6 (0.9)	7.7 (1.6)
**Pt**	0.7 (0.1)	1.9 (0.1)

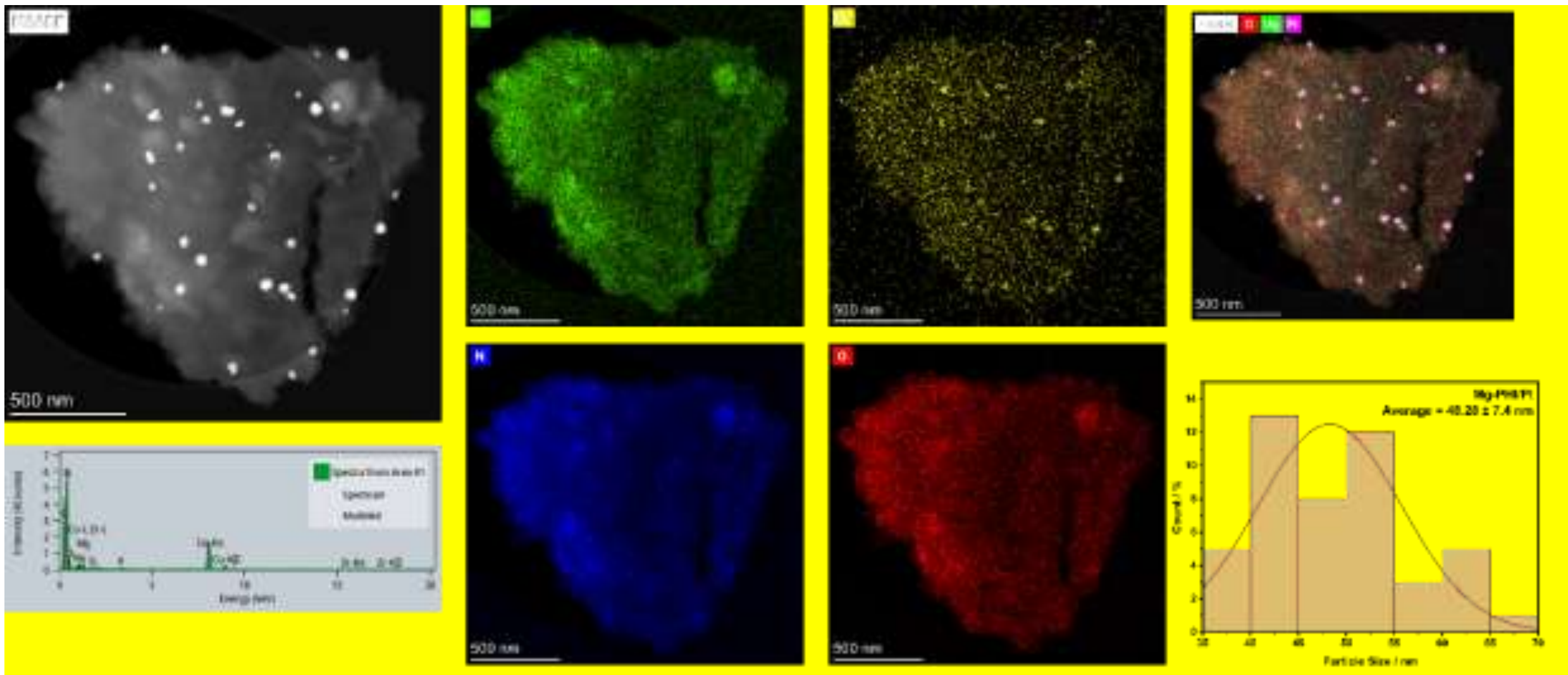

Figure S16. EDX mapping images of the Mg-PHI/Pt photocatalyst. The last panel represents the size distribution of the Pt nanoparticles.

Table S11. Quantitative compositional analysis of Mg-PHI/Pt extracted from TEM.

**Element**	**Atomic Fraction / %**	**Mass Fraction / %**
**C**	42.4 (4.3)	36.8 (3.8)
**N**	49.9 (5.1)	50.5 (5.1)
**O**	4.2 (0.9)	4.9 (1.1)
**Na**	1.5 (0.3)	2.5 (0.6)
**Mg**	1.8 (0.3)	3.1 (0.7)
**Pt**	0.16 (0.1)	2.2 (0.3)

Post-Reaction Characterization

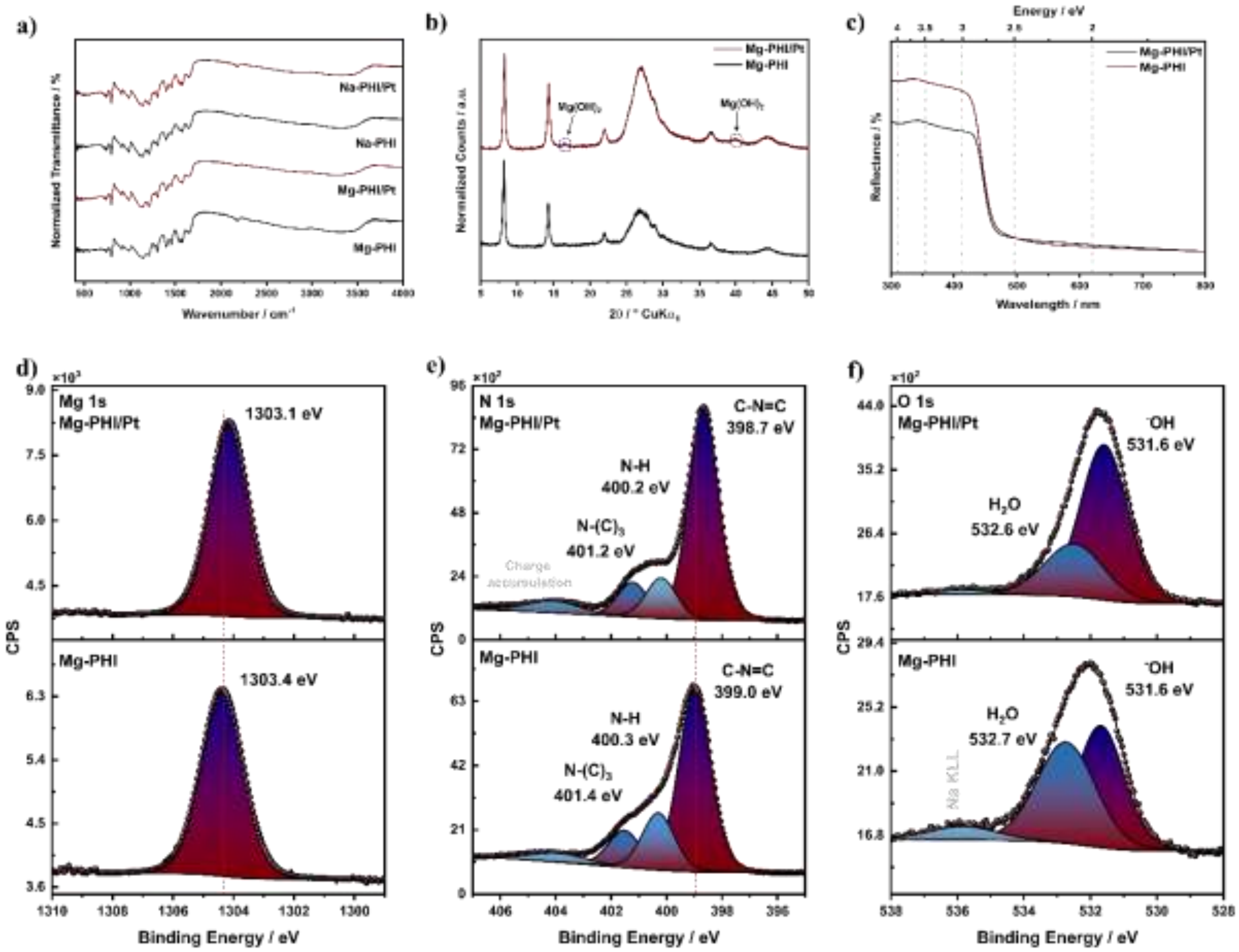

Figure S17. a)FT-IR; **b)** PXRD patterns; **c)** DRS Uv-Vis; **d-f)** HR-XPS Mg 1s, N 1s and O 1s spectra comparison of Na-, Mg-PHI counterpart after the reaction (containing Pt co-catalyst).

Table S12. Quantitative XPS for Mg-PHI/Pt extracted from the survey given in atomic percentage (at%).

O 1s	C 1s	N 1s	Pt 4f	Mg 1s	Na 1s
9.9%	45.4%	42.4%	0.1%	1.3%	0.5%

Molecular Dynamics Simulations

As described in the methodology section, the model for the excited-state simulations reflects the final frame of the MD trajectory, in which the Pt nanoparticle was initially positioned at the central pore. After the MD equilibration for around 150 ns (**Fig. S18a**), the Pt nanoparticle moved to a position between two pores (the central pore and a neighboring pore) with the TEOA molecules completely covering its surface (**Fig. S18b**). This behavior is expected, as the TEOA molecule predominantly contains apolar regions that preferentially interact with the apolar nanoparticle rather than with water, PHI, or Mg^{2+}/Na^{+} cations. Nevertheless, owing to the presence of polar functional groups, the simulations revealed that TEOA molecules can also interact with the cations. In this context, a single TEOA molecule was found in proximity to a cation within the pore and was retained in the model to assess its potential influence on the photoinduced electron-transfer process. Moreover, several water molecules in the vicinity of the nanoparticle surface were retained in the model to evaluate whether photoelectrons can be transferred directly from the nanoparticle to these species. These molecules occupy the voids between the TEOA molecules and establish hydrogen-bonding interactions with them.

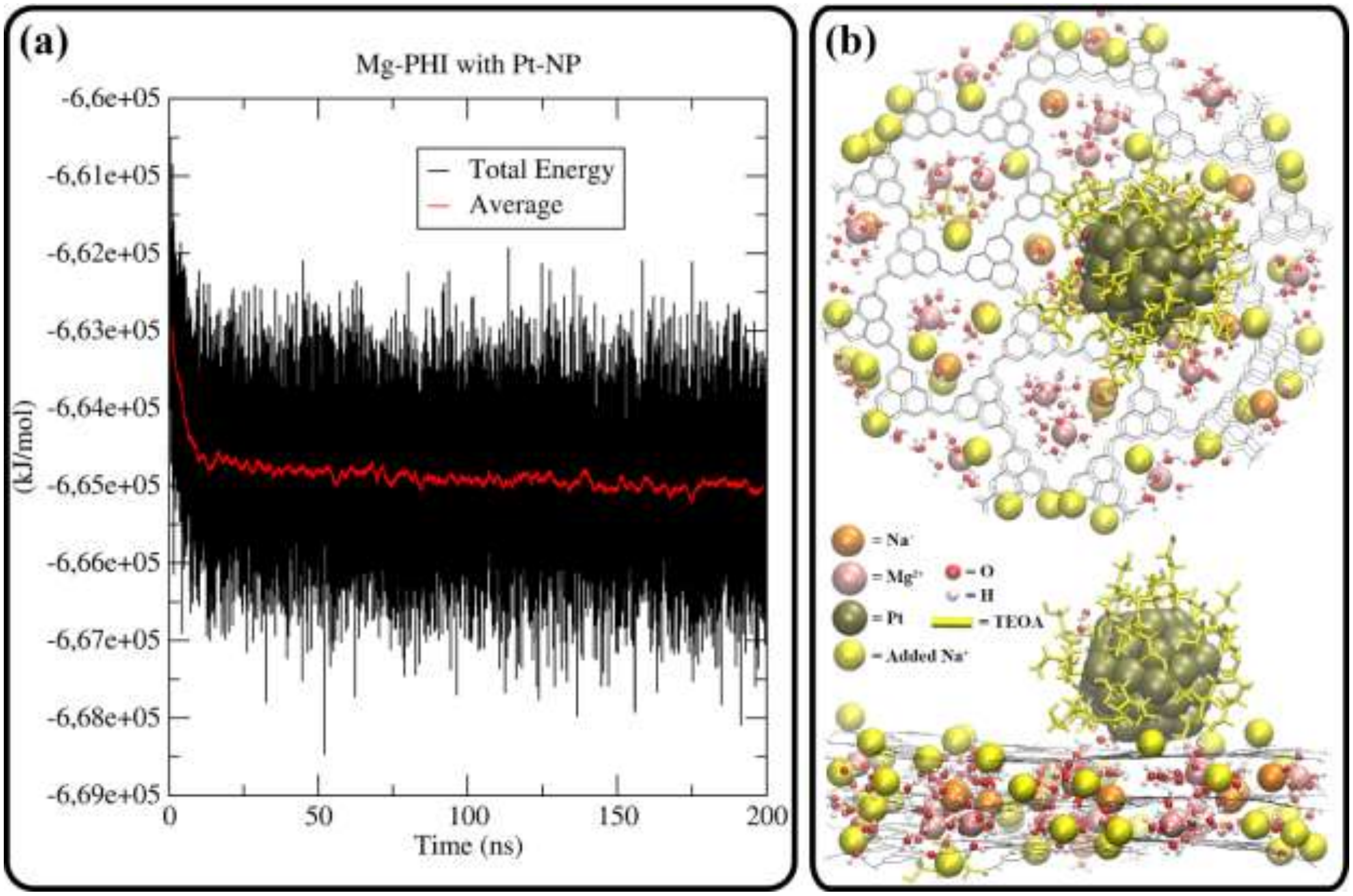

Figure S18. a) Total energy for the MD production run step; **b)** Front (top) and side view (bottom) of the Mg-PHI model used in xTB/sTDA (extended tight binding/simplified Tamm-Dancoff approximation) calculations, with extra 39 Na+ cations added to neutralize the system (yellow spheres).

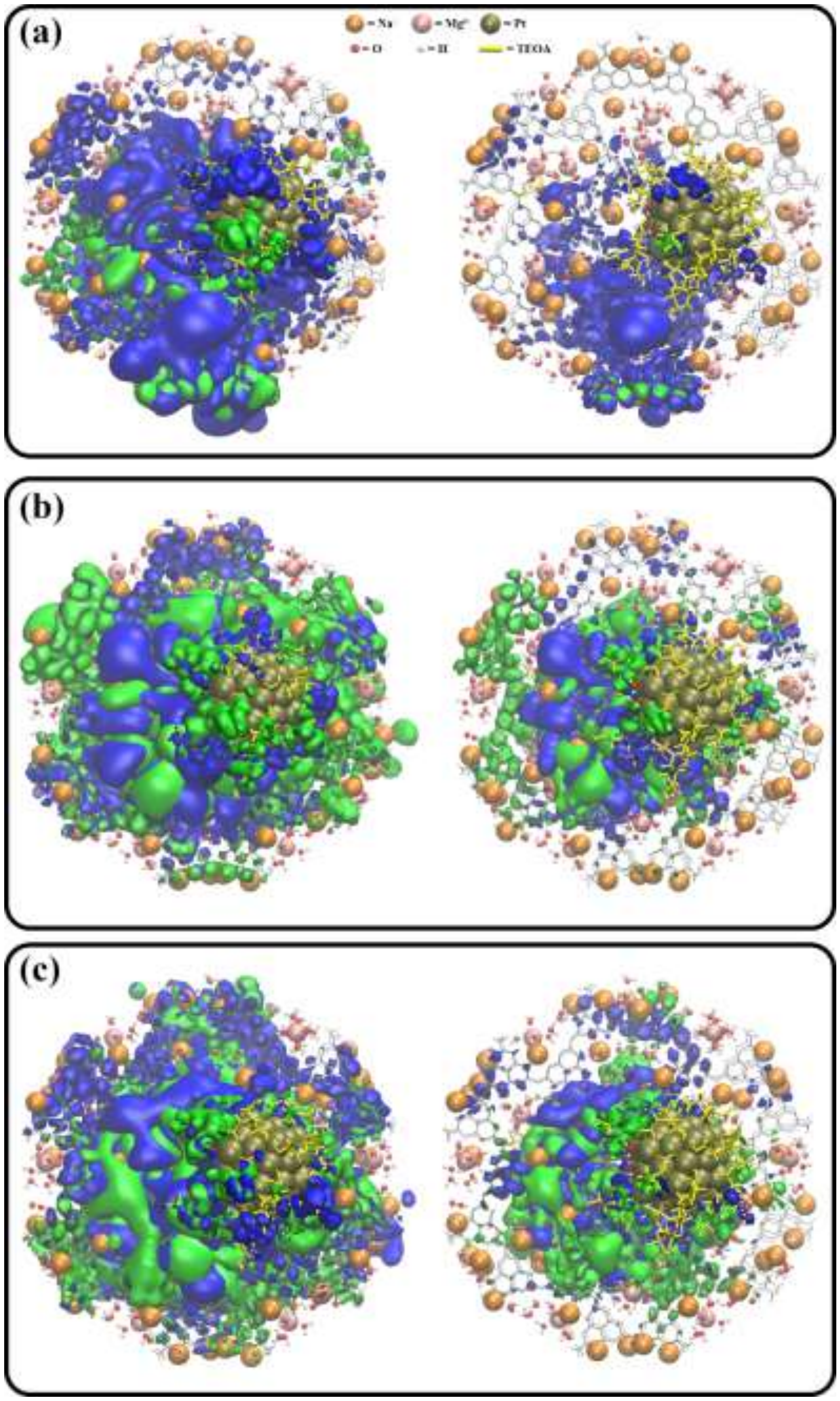

Figure S19. Average NTOs calculated for the transitions with the **a)** second, **b)** third and **c)** fourth highest oscillator strength in the spectral window close to the experimental irradiation wavelength of 410 nm, corresponding to the transitions at 391.1 nm, 403.0 nm and 403.1 nm, respectively. The hole and particle orbitals are depicted as blue and green isosurfaces, respectively, with probabilities of 0.99 (left) and 0.90 (right).

References

(1) Biesinger, M. C. Accessing the robustness of adventitious carbon for charge referencing (correction) purposes in XPS analysis: Insights from a multi-user facility data review. *Applied Surface Science* **2022**, *597*, 153681. DOI: https://doi.org/10.1016/j.apsusc.2022.153681.

(2) Fairley, N.; Fernandez, V.; Richard-Plouet, M.; Guillot-Deudon, C.; Walton, J.; Smith, E.; Flahaut, D.; Greiner, M.; Biesinger, M.; Tougaard, S.; et al. Systematic and collaborative approach to problem solving using X-ray photoelectron spectroscopy. *Applied Surface Science Advances* **2021**, *5*, 100112. DOI: https://doi.org/10.1016/j.apsadv.2021.100112.

(3) Luo, L.; Wang, S.; Zhang, L.; Xiao, X.; Wu, B.; Jaroniec, M.; Jiang, B. Unveiling enhanced dark photocatalysis: Electron storage-enabled hydrogen production in polymeric carbon nitride. *Applied Catalysis B: Environmental* **2024**, *343*, 123475. DOI: https://doi.org/10.1016/j.apcatb.2023.123475.

(4) Wang, Z.; Li, C.; Domen, K. Recent developments in heterogeneous photocatalysts for solar-driven overall water splitting. *Chemical Society Reviews* **2019**, *48* (7), 2109-2125, 10.1039/C8CS00542G. DOI: 10.1039/C8CS00542G.

(5) Colombari, F. M.; Lozada-Blanco, A.; Bernardino, K.; Gomes, W.; de Moura, A. F. Themis: a software to assess association free energies via direct estimative of partition functions. *Unpublished work* **2020**.

(6) Best, R. B.; Zhu, X.; Shim, J.; Lopes, P. E. M.; Mittal, J.; Feig, M.; MacKerell, A. D., Jr. Optimization of the Additive CHARMM All-Atom Protein Force Field Targeting Improved Sampling of the Backbone ϕ, ψ and Side-Chain $\chi 1$ and $\chi 2$ Dihedral Angles. *Journal of Chemical Theory and Computation* **2012**, *8* (9), 3257-3273. DOI: 10.1021/ct300400x.

(7) Berendsen, H. J. C.; van der Spoel, D.; van Drunen, R. GROMACS: A message-passing parallel molecular dynamics implementation. *Computer Physics Communications* **1995**, *91* (1), 43-56. DOI: https://doi.org/10.1016/0010-4655(95)00042-E.

(8) Abraham, M. J.; Murtola, T.; Schulz, R.; Páll, S.; Smith, J. C.; Hess, B.; Lindahl, E. GROMACS: High performance molecular simulations through multi-level parallelism from laptops to supercomputers. *SoftwareX* **2015**, *1-2*, 19-25. DOI: https://doi.org/10.1016/j.softx.2015.06.001.

(9) Berendsen, H. J. C.; Postma, J. P. M.; van Gunsteren, W. F.; DiNola, A.; Haak, J. R. Molecular dynamics with coupling to an external bath. *The Journal of Chemical Physics* **1984**, *81* (8), 3684-3690. DOI: 10.1063/1.448118 (accessed 9/17/2025).

(10) Parrinello, M.; Rahman, A. Polymorphic transitions in single crystals: A new molecular dynamics method. *Journal of Applied Physics* **1981**, *52* (12), 7182-7190. DOI: 10.1063/1.328693 (accessed 9/17/2025).
(11) Hess, B.; Bekker, H.; Berendsen, H. J. C.; Fraaije, J. G. E. M. LINCS: A linear constraint solver for molecular simulations. *Journal of Computational Chemistry* **1997**, *18* (12), 1463-1472. DOI: https://doi.org/10.1002/(SICI)1096-987X(199709)18:12<1463::AID-JCC4>3.0.CO;2-H.
(12) Grimme, S.; Bannwarth, C. Ultra-fast computation of electronic spectra for large systems by tight-binding based simplified Tamm-Dancoff approximation (sTDA-xTB). *The Journal of Chemical Physics* **2016**, *145* (5). DOI: 10.1063/1.4959605 (accessed 9/17/2025).
(13) Bannwarth, C.; Caldeweyher, E.; Ehlert, S.; Hansen, A.; Pracht, P.; Seibert, J.; Spicher, S.; Grimme, S. Extended tight-binding quantum chemistry methods. *WIREs Computational Molecular Science* **2021**, *11* (2), e1493. DOI: https://doi.org/10.1002/wcms.1493.
(14) Allouche, A.-R. Gabedit—A graphical user interface for computational chemistry softwares. *Journal of Computational Chemistry* **2011**, *32* (1), 174-182. DOI: https://doi.org/10.1002/jcc.21600.
(15) Humphrey, W.; Dalke, A.; Schulten, K. VMD: Visual molecular dynamics. *Journal of Molecular Graphics* **1996**, *14* (1), 33-38. DOI: https://doi.org/10.1016/0263-7855(96)00018-5.
(16) Teixeira, I. F.; Tarakina, N. V.; Silva, I. F.; Atta Diab, G. A.; Salas, N. L.; Savateev, A.; Antonietti, M. Improving hydrogen production for carbon-nitride-based materials: crystallinity, cyanimide groups and alkali metals in solution working synergistically. *Journal of Materials Chemistry A* **2022**, *10* (35), 18156-18161, 10.1039/D2TA05571F. DOI: 10.1039/D2TA05571F.
(17) Adler, C.; Selim, S.; Krivtsov, I.; Li, C.; Mitoraj, D.; Dietzek, B.; Durrant, J. R.; Beranek, R. Photodoping and Fast Charge Extraction in Ionic Carbon Nitride Photoanodes. *Advanced Functional Materials* **2021**, *31* (45), 2105369. DOI: https://doi.org/10.1002/adfm.202105369.
(18) Schwinghammer, K.; Mesch, M. B.; Duppel, V.; Ziegler, C.; Senker, J.; Lotsch, B. V. Crystalline Carbon Nitride Nanosheets for Improved Visible-Light Hydrogen Evolution. *Journal of the American Chemical Society* **2014**, *136* (5), 1730-1733. DOI: 10.1021/ja411321s.
(19) Wang, M.; Zhang, Z.; Chi, Z.; Lou, L.-l.; Li, H.; Yu, H.; Ma, T.; Yu, K.; Wang, H. Alkali Metal Cations as Charge-Transfer Bridge for Polarization Promoted Solar-to-H2 Conversion. *Advanced Functional Materials* **2023**, *33* (9), 2211565. DOI: https://doi.org/10.1002/adfm.202211565.

(20) Rono, N.; K., K. J.; S., M. B.; and Nyamori, V. O. A review of the current status of graphitic carbon nitride. *Critical Reviews in Solid State and Materials Sciences* **2021**, *46* (3), 189-217. DOI: 10.1080/10408436.2019.1709414.
(21) Blaskievicz, S. F.; Teixeira, I. F.; Mascaro, L. H. Evaluation of the photoelectrochemical properties of mono and dual single-atom catalysts. *Journal of Solid State Electrochemistry* **2025**, *29* (3), 1025-1034. DOI: 10.1007/s10008-024-06029-8.
(22) Piankova, D. V.; Zschiesche, H.; Tyutyunnik, A. P.; Svensson Grape, E.; da Silva, C. V. C. R.; Guimarães Junior, W. G.; de Moura, A. F.; Reis, I. F.; Diab, G. A. A.; Filho, J. B. G.; et al. Enhancing the Photocatalytic Performance of Carbon Nitrides Through Controlled Local Structure Modification. *Advanced Functional Materials n/a* (n/a), e11389. DOI: https://doi.org/10.1002/adfm.202511389.
(23) Lau, V. W.-h.; Moudrakovski, I.; Botari, T.; Weinberger, S.; Mesch, M. B.; Duppel, V.; Senker, J.; Blum, V.; Lotsch, B. V. Rational design of carbon nitride photocatalysts by identification of cyanamide defects as catalytically relevant sites. *Nature Communications* **2016**, *7* (1), 12165. DOI: 10.1038/ncomms12165.
